# Therapeutical interference with the epigenetic landscape of germ cell tumors: a comparative drug study and new mechanistical insights

**DOI:** 10.1186/s13148-021-01223-1

**Published:** 2022-01-07

**Authors:** Melanie R. Müller, Aaron Burmeister, Margaretha A. Skowron, Alexa Stephan, Felix Bremmer, Gamal A. Wakileh, Patrick Petzsch, Karl Köhrer, Peter Albers, Daniel Nettersheim

**Affiliations:** 1 Department of Urology, Urological Research Laboratory, Translational UroOncology, Medical Faculty and University Hospital Düsseldorf, Heinrich Heine University Düsseldorf, Universitätsstr. 1, 40225 Düsseldorf, Germany; 2 Institute of Pathology, University Medical Centre Göttingen, Göttingen, Germany; 3 Department of Urology, University Hospital Ulm, Ulm, Germany; 4 Genomics & Transcriptomics Laboratory, Heinrich Heine University Düsseldorf, Düsseldorf, Germany; 5 Department of Urology, Medical Faculty and University Hospital Düsseldorf, Heinrich Heine University Düsseldorf, Düsseldorf, Germany

**Keywords:** Germ cell tumor, Therapy, Epi-drugs, Histone modification, Epigenetic modifier, Epigenetics, Histone deacetylase inhibitor, Histone demethylase inhibitor, Histone methyltransferase inhibitor, PROTAC

## Abstract

**Background:**

Type II germ cell tumors (GCT) are the most common solid cancers in males of age 15 to 35 years. Treatment of these tumors includes cisplatin-based therapy achieving high cure rates, but also leading to late toxicities. As mainly young men are suffering from GCTs, late toxicities play a major role regarding life expectancy, and the development of therapy resistance emphasizes the need for alternative therapeutic options. GCTs are highly susceptible to interference with the epigenetic landscape; therefore, this study focuses on screening of drugs against epigenetic factors as a treatment option for GCTs.

**Results:**

We present seven different epigenetic inhibitors efficiently decreasing cell viability in GCT cell lines including cisplatin-resistant subclones at low concentrations by targeting epigenetic modifiers and interactors, like histone deacetylases (Quisinostat), histone demethylases (JIB-04), histone methyltransferases (Chaetocin), epigenetic readers (MZ-1, LP99) and polycomb-repressive complexes (PRT4165, GSK343). Mass spectrometry-based analyses of the histone modification landscape revealed effects beyond the expected mode-of-action of each drug, suggesting a wider spectrum of activity than initially assumed. Moreover, we characterized the effects of each drug on the transcriptome of GCT cells by RNA sequencing and found common deregulations in gene expression of ion transporters and DNA-binding factors. A kinase array revealed deregulations of signaling pathways, like cAMP, JAK-STAT and WNT.

**Conclusion:**

Our study identified seven drugs against epigenetic modifiers to treat cisplatin-resistant GCTs. Further, we extensively analyzed off-target effects and modes-of-action, which are important for risk assessment of the individual drugs.

**Supplementary Information:**

The online version contains supplementary material available at 10.1186/s13148-021-01223-1.

## Background

According to the World Health Organization (WHO) classification system, germ cell tumors (GCT) of the testis can be subdivided into two main groups [1]. One group derives from a pre-cancerous, non-invasive disease named germ cell neoplasia in situ (GCNIS), and is classified as type II GCT, which occur primarily after puberty in young males of age 15—35 years. The second GCT group (type I and III GCT) does not originate from GCNIS and affects prepubertal children and infants (type I) or older men (type III, spermatocytic tumors), respectively [1–3]. Type II GCTs can be stratified into seminomas (SE) and non-seminomas and present as pure or intermixed subtypes. The stem cell-like non-seminomatous embryonal carcinomas (ECs) can further differentiate into teratomas (TE) and extra-embryonic tissues, i.e. yolk-sac tumors (YSTs) and choriocarcinomas (CCs) [2–4].

Even though cure rates are up to 90%, mostly due to cisplatin based therapy, the incidences keep rising [4]. Additionally, 10–15% of patients relapse and develop therapy resistance with poor survival rates [5]. Therefore, new therapeutic approaches to treat cisplatin refractory GCTs and circumvent late toxicities induced by the cisplatin-based therapy are needed [6].

Compared to most other cancer types, GCTs harbor a very low mutational burden [7, 8]. Hence, it is postulated that epigenetic aberrations could be the driver of tumorigenesis in GCTs [3, 9]. It has been shown that the GCT subtypes vary greatly in their DNA methylation status, i.e. hypomethylation in SE and hypermethylation in non-seminomas [8, 10]. Little is known about histone modifications in GCT, but a few studies and review articles point out that GCTs in general may have high levels of bivalent histone marks H3K27me3 and H3K4me3 [11, 12]. Almstrup et al. detected high levels of H3K9me2, H3K27me3 and H3K4me1 and low levels of H3K4me2/3 in SE, and vice versa in EC [13]. Further, high levels of symmetric arginine dimethylation (H2AR3me2 and H4R3me2) were found in SE, while low levels were measured in EC [14]. Although studies analyzing the genome wide histone modification landscape in GCTs are lacking, analyses of expression levels of epigenetic modifiers and related drug studies are available [10, 15–17].

Previously, we described the cytotoxic effects of the histone deacetylase (HDAC) inhibitor Romidepsin [16, 18] and bromodomain-containing protein (BRD) inhibitor JQ1 [15], which both target modulators of the epigenetic landscape. Both inhibitors caused considerable transcriptional changes, affected the cell cycle and induced apoptosis in vitro as well as in vivo, resulting in significantly reduced tumor growth in mouse xenografts [15, 16]. As these studies showed promising results in using epigenetic inhibitors as a treatment option for GCTs, we further analyzed different drugs against epigenetic modifiers and readers, which influence histone and DNA methylation, histone acetylation and histone ubiquitination, but are not approved for the treatment of cancerous malignancies, yet. Therefore, this study investigated various epigenetic drugs (epi-drugs) and discussed their potential as new therapeutic options for refractory GCTs.

## Results

### Identifying epi-drug targets in GCTs

To identify *bona fide* epigenetic targets in GCTs, we re-analyzed microarray expression data of different epigenetic writers, erasers and readers in the GCT entities GCNIS, SE, EC and teratoma (Additional file 1: Fig. S1A) [10, 19]. From the 127 analyzed targets, 80 were commonly and significantly expressed (expression intensity > log_2_10) among the different GCT entities (Additional file 1: Fig. S1B). Next, these 80 targets were checked for expression in GCT cell lines to allow for in vitro screening of potential drugs (Additional file 1: Fig. S1C, D) [15, 16, 20–22]. 33 epigenetic modulators were commonly expressed among the cell lines (SE: TCam-2; EC: 2102EP, NCCIT; CC: JAR) (Additional file 1: Fig. S1C, D). We identified 14 inhibitors against these targets, including histone deacetylase inhibitors (HDACi), histone demethylase inhibitors (HDMi), histone methyltransferase inhibitors (HMTi), inhibitors of proteins binding to acetylated histones (PAHi), polycomb-repressive complex inhibitors (PRCi) and DNA methyltransferase inhibitors (DNMTi). Inhibitors were chosen based on a high specificity for their respective target (Additional file 1: Fig. S1E) [23–36]. Further, two proteolysis targeting chimera (PROTACs) were also included in this study, i.e., VZ185 targeting BRD7/BRD9 and MZ-1 targeting BRD4 [37, 38].

Of note, mRNA levels of *BRD4* and *EZH2* were quite low in GCT tissues and cell lines (Additional file 1: Fig. S1A, D). Nevertheless, previous studies on the bromodomain and extra-terminal motif (BET)/BRD4 inhibitor JQ1 detected high levels of BRD4 on protein level in GCT cell lines [15]. A probable explanation for this observation could be the (epigenetic) regulation and activity of BRD proteins. Elevated expression of *BRD4* in pancreatic cancer has been attributed to presence of 5-hydroxymethylcytosine (5hmC) at the *BRD4* promoter, indicative of an active DNA demethylation process (ADD) [39]. Moreover, based on ‘methylation-inscribed nascent transcripts sequencing’ (MINT-seq), N^6^-methyladenosine (m^6^A)-marked enhancer RNAs (eRNAs) have been identified to recruit the nuclear m^6^A reader YTHDC1, resulting in the formation of m^6^A-eRNA/YTHDC1 condensates, which promote *BRD4*/BRD4 co-activator formation [40]. Further, Cheung et al. summarized that the expression and activity of *BRD4* was further regulated by miRNA binding to the 3′ UTR of *BRD4*, as well as post-translationally by acetylation, phosphorylation, ubiquitination and proline hydroxylation [41]. Additionally, we validated high BRD4 and EZH2 levels by western blotting in four different GCT cell lines (Additional file 1: Fig. S1F). Furthermore, we detected the VHL-E3 ubiquitin ligase by western blotting (Additional file 1: Fig. S1F) [37, 38]. In conclusion, in GCTs both BRD4 and EZH2 can be targeted by related epi-drugs and PROTACs, which depend on presence of VHL-E3.

**Fig. 1:**
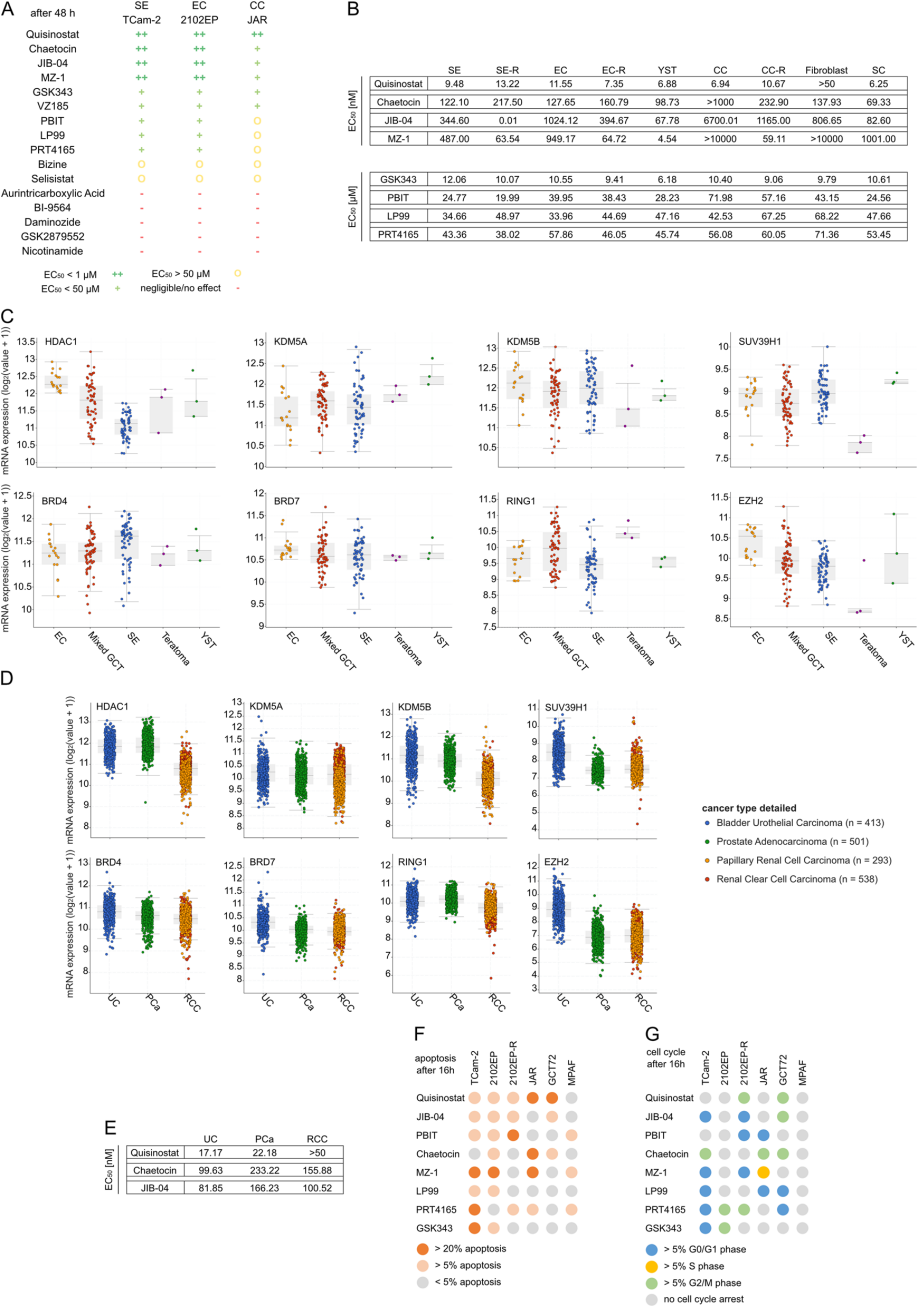
8 out of 16 drugs were able to reduce cell viability in GCT cell lines. **A** Table of the initial 16 drugs tested in 3 GCT cell lines. The color/symbol code is based on the EC_50_ after 48 h of treatment as determined by XTT assay. See also Additional file 2: Fig. S2 (*n* = 4). **B** List of the mean EC_50_ values per GCT entity, as well as fibroblasts and Sertoli cells, after 48 h. See also data in Additional file 3: Fig. S3. For single values, see Additional file 4: Fig. S4A. **C** Gene expression profiles of the TCGA 'Testicular Germ Cell Tumors' cohort for each target gene. **D** Expression analysis of TCGA data for each target gene in the urological tumor entities UC, PCa and RCC. **E** Mean EC_50_ values of three cell lines from UC, PCa and RCC. For raw data, see Additional file 4: Fig. S4C–E. For single values, see Additional file 4: Fig. S4F (*n* = 4). **F**, **G** Flow cytometric analysis of apoptosis (**F**) and cell cycle (**G**) after 16 h. Only changes over 5% compared to solvent control were considered significant. See also Additional file 5: Fig. S5. EC_50_: half-maximal effective concentration where the cell viability has 50% compared to the solvent control; SE, seminoma; EC, embryonal carcinoma; YST, yolk-sac tumor; CC, choriocarcinoma; SC, Sertoli cells; -R, cisplatin-resistant subclone; UC, urothelial carcinoma; PCa, prostate cancer; RCC, renal cell carcinoma

In total, 16 epi-drugs were selected (Table 1). In a first pre-screen, we assessed the cytotoxic effects in three GCT cell lines (SE: TCam-2, EC: 2102EP, CC: JAR) and in non-malignant fibroblast cells MPAF (Fig. 1A; Additional file 2: Fig. S2). XTT cell viability assays revealed that four drugs (Quisinostat, Chaetocin, JIB-04 and MZ-1) were most efficient in reducing cell viability in SE and EC proxies TCam-2 and 2102EP with EC_50_ values (half-maximal effective concentration) in a nanomolar range, while GSK343, PBIT, PRT4165 and LP99 showed EC_50_ concentrations up to 50 µM. Additionally, VZ185, a recently published PROTAC [38], was tested effectively at low micromolar concentrations, further validating BRD7/BRD9 as a suitable target. Due to low cytotoxic effects in the first screening approach (EC_50_ > 50 µM), Selisistat, Nicotinamide, GSK2879552, Bizine, BI-9564 and Aurintricarboxylic Acid were excluded from further analyses. Generally, all drugs were less effective in targeting JAR cells (CC) as well as in the MPAF fibroblast cells (mean EC_50_ value 44.5 µM and 25.4 µM, respectively) (Additional file 2: Fig. S2).

**Table 1:** List of epi-drugs used in this study

Type	Target	Drug	References
HDACi	HDAC1	Quisinostat	[23]
	SIRT1	Selisistat, Nicotinamide	[25, 28]
HDMi	KDM1A/LSD1/AOF2	GSK2879552, Bizine	[30, 33]
	KDM2A/FBXL11	Daminozide	[32]
	KDM5A/JARID1A	JIB-04	[35]
	KDM5B/JARID1B	PBIT	[26]
HMTi	SUV39H1	Chaetocin	[31]
PAHi	BRD4	MZ-1	[37]
	BRD7	LP99, VZ185	[36, 38]
	BRD9	LP99, VZ185, BI-9564	[29, 36, 38]
PRC1i	RING1	PRT4165	[27]
PRC2i	EZH2	GSK343	[34]
DNMTi	DNMT1	Aurintricarboxylic acid	[24]

Type of target, target name, drug name and reference are indicated. HDACi: histone deacetylase inhibitor, HDMi: histone demethylase inhibitor, HMTi: histone methyltransferase inhibitor, PAHi: inhibitor of proteins binding to acetylated histones, PRCi: polycomb-repressive complex inhibitor, DNMTi: DNA methyltransferase inhibitor

The effect on cell viability of the most efficient eight drugs was then extended to a broader range of cell lines including NCCIT, NT2/D1 (EC), JEG-3, BeWo (CC) and GCT72, 1411H (YST), their cisplatin-resistant sublines (-R), as well as another fibroblast cell line (HVHF2) and Sertoli cells FS1 (Fig. 1B; Additional file 3: Fig. S3, Additional file 4: Fig. S4A). The SE, EC and YST cell lines responded well to the treatment with all tested drugs (except SE in GSK343 treatment) (Fig. 1B). The concentrations required to reduce cell viability were lower in GCTs compared to fibroblasts (MPAF, HVHF2), and in a few cases also compared to Sertoli cells (FS1) (Fig. 1B, Additional file 4: Fig. S4A). In general, the three CC cell lines responded heterogeneously to the epi-drug treatments, e.g. EC_50_ values upon Chaetocin treatment were >1000 nM, 478.4 nM and 126.6 nM or upon MZ-1 treatment were >10,000 nM, 83.0 nM and 5261 nM, respectively.  In general, BeWo showed a similar sensitivity towards the epi-drugs as SE and EC cell lines (EC_50_ values ranging from 5.2 nM to 41.7 µM, 9.5 to 43.4 µM and 11.6 nM to 57.9 µM, respectively), while JAR and JEG-3 were less responsive to the epi-drugs (EC_50_ values ranging from 6.12 nM to >100 µM and 9.5 nM to 72.8 µM, respectively) (Additional file 4: Fig. S4A).

Next, gene expression and mutation profiles of the targets of the eight epi-drugs with highest efficiency (Quisinostat, JIB-04, PBIT, Chaetocin, MZ-1, LP99, PRT4165, GSK343) were analyzed by screening the 'Testicular Germ Cell Tumors' cohort of ‘The Cancer Genome Atlas’ (TCGA [8, 42, 43]) using cBioPortal [44, 45] (Fig. 1C, Additional file 4: Fig. S4B). Overall, mutations were rare in the selected target genes in the 144 GCT samples, with the highest mutation rate of 6% found in *KDM5A* (JIB-04 target) (Additional file 4: Fig. S4B). Differences in mRNA expression levels were more common in all GCT types, e.g. high mRNA levels of *EZH2* (GSK343 target) and *HDAC1* (Quisinostat target) were observed in 17% and 13% of the samples, respectively. All target genes were expressed in seminomatous and non-seminomatous GCT (with lowest mRNA levels of *SUV39H1* in YST), further corroborating the potential use of the selected drugs (Fig. 1C).

In addition, other urological malignancies (urothelial carcinoma (UC), prostate cancer (PCa) and renal cell carcinoma (RCC)) were screened for the expression of the target molecules of Quisinostat, JIB-04, PBIT, Chaetocin, MZ-1, LP99, PRT4165 and GSK343 (Fig. 1D). The target genes were also expressed in these entities, except for *EZH2* (GSK343), which was marginally expressed in PCa and RCC. Based on these data, the three most potent drugs Quisinostat, JIB-04 and Chaetocin were tested in UC, PCa and RCC cell lines (Fig. 1E, Additional file 4: Fig. S4C–F). Quisinostat was less efficient in these entities than in GCT cell lines, but still required only low nanomolar concentrations to kill the cells (EC_50_ values 17.2 nM, 22.2 nM and 44.3 nM in UC, PCa and RCC, respectively). Similar to the observations found in GCTs, JIB-04 and Chaetocin were highly effective in these urological entities, thereby highlighting the pan-urologic applicability of these epi-drugs.

### Epi-drugs induce apoptosis and a cell cycle arrest

Next, we analyzed whether the reduced cell viability caused by the epi-drugs resulted from an induction of apoptosis or a cell cycle arrest. Treatments with the epi-drugs (EC_50_, 16 hours (h)) induced apoptosis in the majority of the tested GCT cell lines, with TCam-2 and 2102EP being affected the most (Fig. 1F, Additional file 5: Fig. S5). Interestingly, apoptosis induction was negligible in JAR and GCT72 upon treatment with PBIT, LP99 and GSK343. Additionally,the fibroblast control cells showed only a weak apoptosis induction upon treatment with PBIT, MZ-1 and PRT4165. The cisplatin-resistant subline of 2102EP (2102EP-R) did not induce apoptosis after treatment with Chaetocin, MZ-1, LP99 or GSK343 compared to the parental cell line, while MZ-1 treatment induced a cell cycle arrest in 2102EP-R (Fig. 1F, G). Generally, the cell cycle phase distribution was affected upon epi-drug treatment in a cell line-dependent manner (Fig. 1G, Additional file 5: Fig. S5). Quisinostat or Chaetocin treatment resulted in an accumulation in the G2/M phase, while GCT cells treated with PBIT, LP99 and partly MZ-1 accumulated in the G0/G1 phase. In contrast, no changes in the cell cycle phase distribution were observed in MPAF fibroblast cells (Fig. 1G). Hence, in GCTs treatment with epi-drugs induced both, apoptosis and cycle arrest in a cell line-dependent manner, while epi-drugs were generally less effective in non-cancerous fibroblasts.

Due to high target similarity of the inhibitors JIB-04 and PBIT (Jumonji histone demethylases KDM5A and KDM5B), PBIT was excluded from further molecular analysis due to its higher EC_50_ values compared to JIB-04.

### Treatment with epi-drugs result in epigenetic off-target effects

**Fig. 2:**
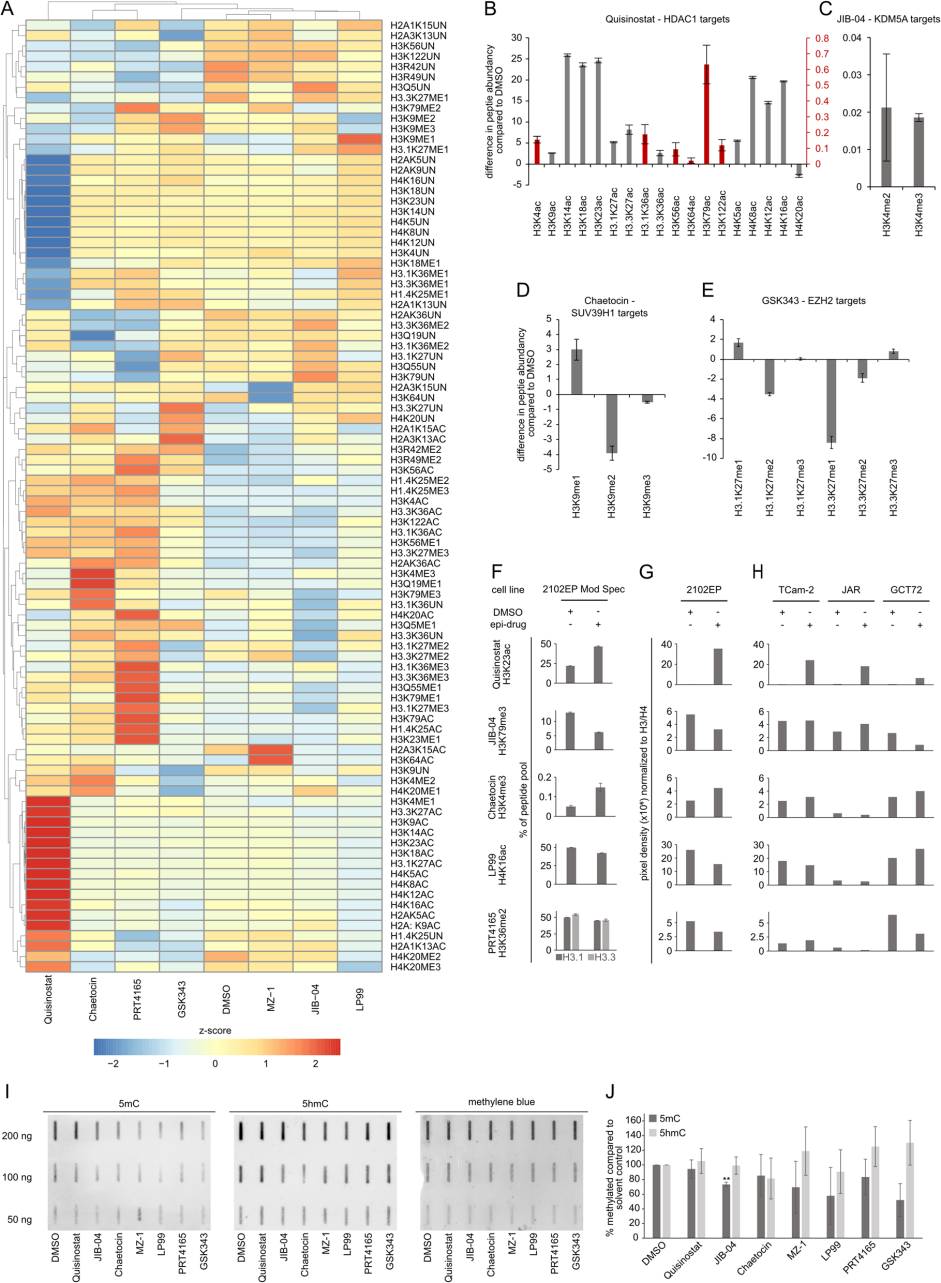
Global analysis of the histone modification landscape revealed off-target effects of each epi-drug. **A** An unsupervised hierarchical clustering heatmap summarizes the results of the mass spectrometry analysis (Mod Spec) of 2102EP cells treated for 16 h with indicated epi-drugs (*n* = 3). **B**–**E** The bar charts show the differences in peptide abundancy for histone modifications of the inhibitor target molecules HDAC1 (Quisinostat (**B**)), KDM5A (JIB-04 (**C**)), SUV39H1 (Chaetocin (**D**)) and EZH2 (GSK343 (**E**)). For raw data, see Additional file 11: Table S1 A (technical replicates *n* = 3). **F** Detected changes at indicated histone modifications as found by Mod Spec analysis. **G** Validation of changes given in (**F**) using western blot analysis. Data was normalized to total H3 or H4 and densitometrically evaluated. Cells were treated with indicated epi-drugs for 16 h. For raw data, see Additional file 6: Fig. S6F. **H** Analysis of additional GCT cell lines for changes in the same modifications as given in (**F**, **G**). For raw data, see Additional file 6: Fig. S6F. **I** DNA methylation analysis using the slot blot technique. 2102EP cells were treated with indicated epi-drugs for 24 h. A separate membrane was used for each detection. A dilution series (*n* = 3) was prepared for the three membranes at once. Methylene blue staining served as loading control. **J** Densitometric analysis of the slot blot membranes. Pixel density of 5mC and 5hmC blots was normalized to methylene blue staining. Changes in DNA methylation are given as percentage of solvent control (set to 100%). Statistical analysis was performed using paired *t* test (*p < 0.05, **p < 0.005)

To screen for the effects of each epi-drug on the epigenetic landscape, mass spectrometry analyses of histone modifications were performed in the EC cell line 2102EP after treatment with Quisinostat, JIB-04, Chaetocin, MZ-1, LP99, PRT4165 or GSK343, allowing to not only confirm known changes in the epigenetic landscape caused by each epi-drug, but also to identify epigenetic effects beyond the postulated modes of action (Fig. 2A, Additional file 11: Table S1 A, B). Questioned histone modifications were categorized into three groups of different peptide abundancies to enable an unbiased comparison of changes (Additional file 6: Fig. S6A, D). Especially the less frequent histone modifications (peptide frequency < 10%) accumulated in response to used inhibitors. As expected, HDACi Quisinostat induced a strong accumulation of histone acetylation on histones H3 and H4, apart from H4K20ac and H2A3K15ac, which showed a decreased frequency (Fig. 2A, B; Additional file 6: Fig. S6A–C). In addition, off-target effects, such as an increase in histone methylation (H3K4me1, H3.3K27me3, H3K56me1, H4K20me1/3) were noted (Additional file 6: Fig. S6A–C). JIB-04, a HDMi primarily targeting KDM5A/JARID1A and specifically removing methyl groups from di- and trimethylated H3K4 [46], slightly increased H3K4me2/me3 marks (Fig. 2A, C; Additional file 6: Fig. S6C). Treatments with the HMTi Chaetocin and GSK343 reduced methylation at the respective histones. The inhibition of the H3K9-di/tri-methyltransferase SUV39H1 [47, 48] (Chaetocin) led to a reduction of H3K9me2/me3, while H3K9me1 increased (Fig. 2A, D, Additional file 6: Fig. S6A, B). Inhibition of EZH2/PRC2 resulted in a decreased H3K27 methylation [49], with the exception of H3.1K27me1 and H3.3K27me3 (slightly increased) as well as H3.1K27me3 (unchanged) (Fig. 2A, E; Additional file 6: Fig. S6A, B). Changes in histone acetylation were observed as off-target effects of HDMi and HMTi treatments. As such, JIB-04 treatment led to a decrease in H4K16ac and H4K20ac, whereas both, Chaetocin and GSK343 treatment, led to an increase of H4K20ac (Additional file 6: Fig. S6A). Also, off-target changes in histone methylation could be detected (e.g. JIB-04: H3K27me2, H3K79me3; Chaetocin: H3K4me2/me3, H4K20me1/me2/me3; GSK343: H3K4me2, H4K20me2) (Fig. 2, Additional file 6: Fig. S6A–C). For PRT4165 treatment, a PRC1/RING1 inhibitor, a decreased ubiquitination (ub) of the target histone H2AK119 [50] could not be detected. However, off-target effects were also observed for PRT4165, e.g. an increase of H3K36me3, H4K20ac, H3K79me1/ac (Fig. 2A; Additional file 6: Fig. S6A, C). Surprisingly, the two drugs targeting readers of histone acetylation (MZ-1, LP99) also led to alterations in histone modifications. BRD7/9 inhibitor LP99 led to a general decrease in histone acetylation, alongside a few increases in methylation (H3K9me1, H3K27me1, H3K36me1) (Fig. 2A; Additional file 6: Fig. S6A). The PROTAC MZ-1 showed the highest similarity to the solvent control as there were only two modifications significantly altered (increase in H3K64ac and H2A3K15ac) (Additional file 2: Fig. S2A). H3R42me2 and H3R49me2 were found to be commonly increased upon all epi-drug treatments (Additional file 5: Fig. S5B).

By western blot analyses, a decreased H2AK119ub could be confirmed after 48 h of PRT4165 treatment (Additional file 6: Fig. S6E). To further validate the mass spectrometry data, western blot analyses of selected on- as well as off-targets were performed using epi-drug treated 2102EP cells (Fig. 2F, G; Additional file 6: Fig S6F). Some changes observed in 2102EP could be mirrored to other cell lines (TCam-2 (SE), JAR (CC) and GCT72 (YST)), like the increase of histone acetylation upon Quisinostat treatment (Fig. 2H, Additional file 6: Fig. S6F). Analyzed off-target changes were not commonly altered among all tested cell lines (showing similarities in max. two other cell lines).  The highest similarity was observed between EC (2102EP) and YST (GCT72) cells, reacting differently only towards LP99 treatment.

To test, whether interference with histone interacting factors also had an impact on DNA methylation, slot blot analyses were performed using DNA of 2102EP cells treated for 24 h with different epi-drugs (Fig. 2I, J). All tested epi-drugs decreased 5-methylcytosine (5mC) methylation levels (except for Quisinostat) compared to solvent control. Lowest 5mC levels were found after LP99 treatment (58% compared to solvent control). JIB-04 treatment led to a significant reduction in 5mC levels (27%). Chaetocin, MZ-1, PRT4165 and GSK343 reduced the 5mC levels to 85%, 70%, 84% and 52% compared to solvent control, respectively. 5hmC (as part of the active DNA demethylation cascade) levels increased upon MZ-1 (19%), PRT4165 (25%), GSK343 (30%) treatment compared to solvent control. In conclusion, we could confirm expected changes upon epi-drug treatments, but also observed several cell line-dependent off-target histone modifications and changes in the DNA-methylation landscape.

### Interfering with the epigenetic landscape results in deregulation of transcriptional regulators

**Fig. 3:**
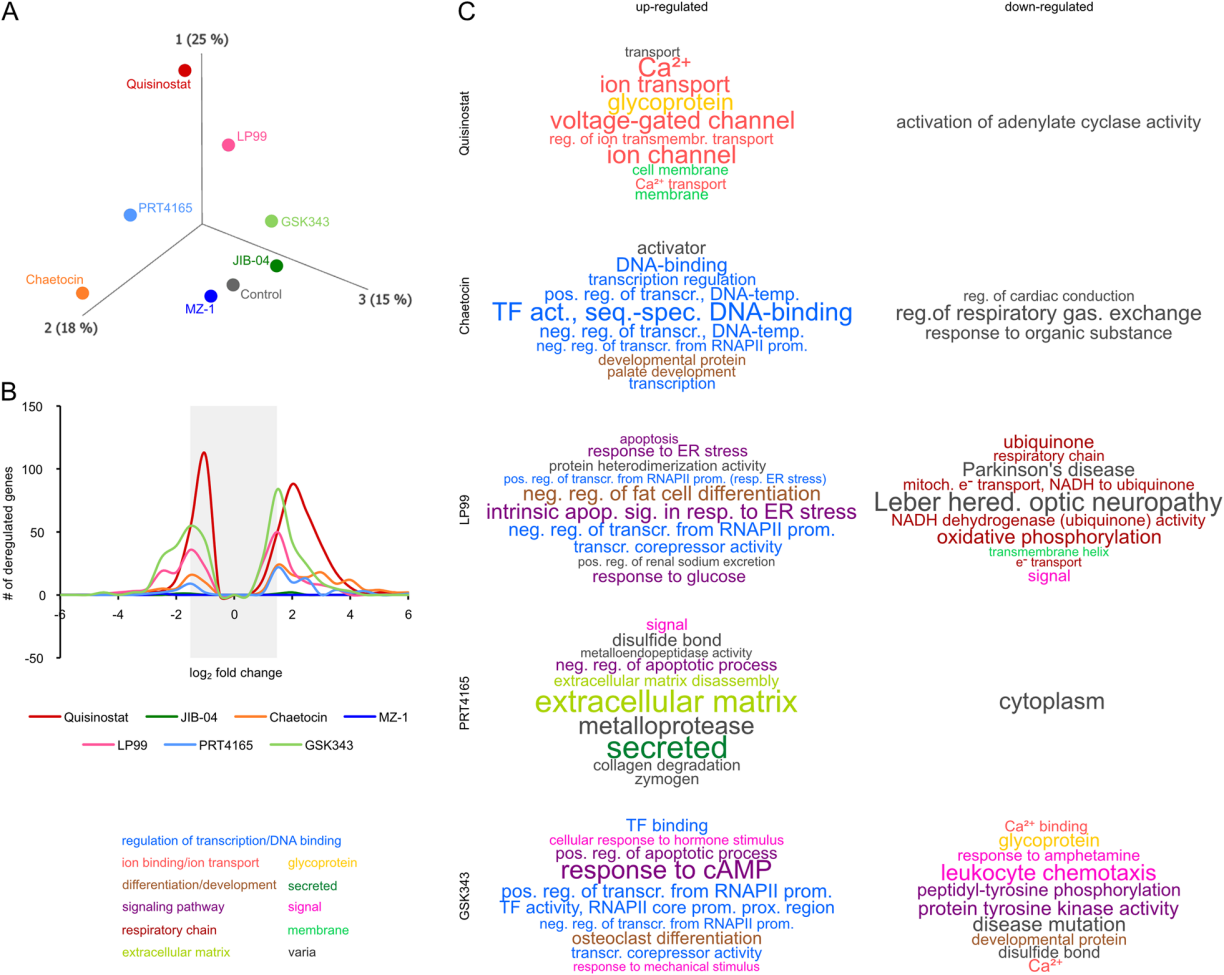
Epi-drugs induced deregulations of genes associated with glycoproteins, ion transporters and DNA-binding factors. **A** PCA of the RNA seq data performed in 2102EP 16 h after treatment with the epi-drugs (DMSO control *n* = 3, epi-drugs *n* = 1). **B** Diagram illustrating the number of deregulated genes and correlating fold-changes in gene expression after each epi-drug treatment. Only deregulated genes with an FDR corrected *p* value of < 0.05 were considered. The gray bar indicates genes below the fold change of log_2_ 1.5. **C** Word clouds representing the ten most significant functional annotations of up- or downregulated gene ontologies (filtered by uncorrected *p* value < 0.05). Letter sizes correlate with − log_10_ (*p* value). For more data, see Additional file 12: Table S2 B–H

To gain insights into the transcriptional deregulations caused by disturbances of the epigenetic landscape, a RNA sequencing (RNA seq) analysis was performed in 2102EP EC cells treated with Quisinostat, JIB-04, Chaetocin, MZ-1, LP99, PRT4165 or GSK343 (Fig. 3). Aprincipal component analysis (PCA) and an unsupervised hierarchical clustering heatmap demonstrated that JIB-04 and MZ-1 showed the highest similarity to the solvent controls, while Quisinostat treatment led to the strongest differences on transcriptome level (Fig. 3A; Additional file 7: Fig. S7A). Using a cut-off with an FDR corrected *p* value of ≤ 0.05, we identified 474 genes, which were altered in expression after Quisinostat treatment. In general, the treatment with epi-drugs could be associated with an increased transcriptional activity, since more genes were upregulated than downregulated (Fig. 3B). Only negligible transcriptional changes were observed upon MZ-1 and JIB-04 treatment (Fig. 3B).

**Fig. 4:**
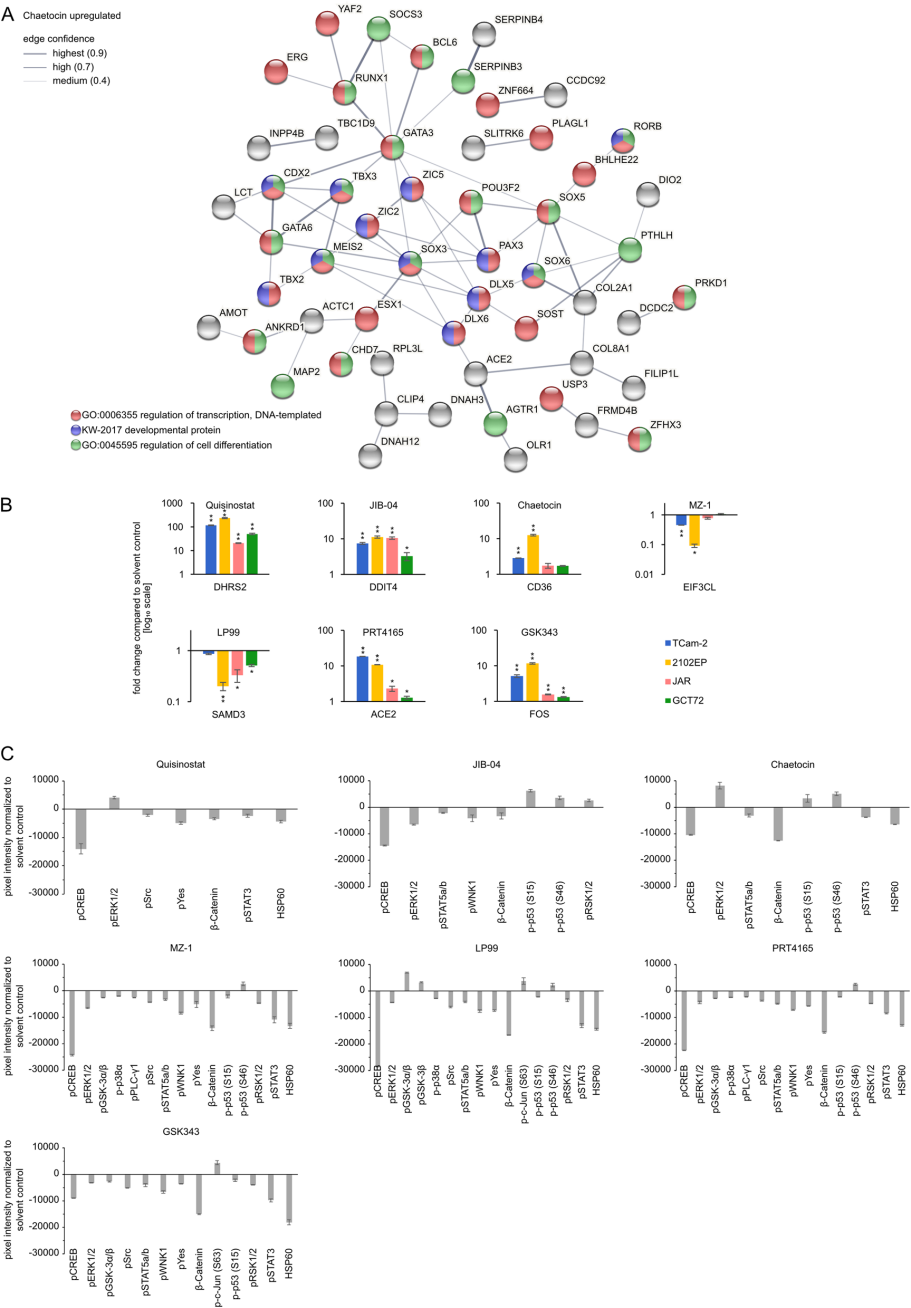
Epi-drug treatments induced deregulations in expression of transcriptional regulators and changes in kinase signaling pathways. **A** STRING interaction prediction of upregulated genes in Chaetocin treated (16 h) 2102EP cells. **B** qRT-PCR validation of selected genes in four GCT cell lines (*n* = 3). Asterisks indicate significant changes between treatment and solvent control (*p < 0.05, **p < 0.005). **C** Densitometric analysis of signals from the human phospho-kinase array membranes (*n* = 2); normalized to solvent control of each tested drug. Cells were treated for 16 h with indicated epi-drugs. See also Additional file 9: Fig. S9 A–D. p: phosphorylated protein.

For subsequent functional annotation analysis, only genes with a log_2_ fold change of at least ± 1.5 and an FDR corrected *p* value of ≤ 0.05 were considered as significant (Additional file 11: Table S1 C–I). This resulted in a set of 243 significantly deregulated genes for Quisinostat treatment (223 up- and 20 downregulated). The functional annotation of upregulated genes  involved ‘ion transport’ and ‘cell membrane compartments,’ whereas genes involved in ‘activation of adenylate cyclase activity’ were downregulated (Fig. 3C; Additional file 11: Table S1 C). A STRING analysis  further demonstrated interaction of genes involved in ‘ion transport’ and ‘development of the nervous system’ (Additional file 7: Fig. S7B). The HDMi JIB-04 significantly affected expression of only three genes: upregulation of *SLC16A3* (monocarboxylate transporter 4) and *DDIT4* (DNA damage-inducible transcript 4) and downregulation of *EIF3CL* (eukaryotic translation initiation factor 3 subunit C-like) (Additional file 11: Table S1 D). No functional annotation or STRING-based interactions could be predicted for JIB-04. Treatment with the HMTi Chaetocin (82 up-, 14 downregulated genes) led to upregulation of genes associated with ‘DNA binding’ (e.g., *DLX5, DLX6, SOX5, GATA3, GATA6, POU3F2*) as well as ‘cell differentiation regulators’, while downregulated genes were involved in ‘regulation of respiratory gas’ and ‘response to organic substance’ (Fig. 3C, Fig. 4A, Additional file 11: Table S1 E). For MZ-1, only *EIF3CL* was found to be significantly downregulated (Additional file 11: Table S1 F). LP99 (43 up-, 48 downregulated genes) led to upregulation of factors involved in the ‘intrinsic apoptotic signaling in response to endoplasmic reticulum stress’ (*CEBPB, DDIT3, TRIB3*) and ‘negative regulation of transcription’ (e.g., *DDIT3, JDP2, VEGFA*) (Fig. 3C, Additional file 11: Table S1 G), while network analysis further affirmed involvement of factors related to ‘apoptosis’ (Additional file 8: Fig. S8A). The downregulated genes included factors associated with ‘electron transport’ and mitochondrial genes from the ‘oxidative phosphorylation’ process (*MT-ATP6/8, MT-ND2/3/4/4L*) (Fig. 3C; Additional file 8: Fig. S8B; Additional file 11: Table S1 G). Treatment with the PRC1 inhibitor PRT4165 led to upregulation of 35 genes linked to ‘secreted factors’ (*CP4A*, *ANXA1*, matrix metalloproteinases *MMP1/3/10*) and the ‘extracellular matrix,’ while 7 downregulated genes were found to be related to ‘cytoplasm’. Furthermore, the STRING algorithm  predicted interaction between the  development- and pluripotency-associated genes *DPPA3* and *DPPA5* (Fig. 3C, Additional file 8: Fig. S8C, D; Additional file 11: Table S1 H). Finally, GSK343 treatment resulted in upregulation of 84 genes involved in ‘transcription factor binding’ and ‘apoptotis’ (*e.g., FOSL1/2, DUSP1, GADD45A, GADD45B, RHOB*) (Fig. 3C; Additional file 8: Fig. S8 E; Additional file 11: Table S1 I). 78 genes were downregulated after GSK343 application, which could be linked to annotations like ‘developmental protein’ and ‘protein tyrosine kinase activity’ (Fig. 3C; Additional file 8: Fig. S8F; Additional file 11: Table S1 I).

A qRT-PCR analysis was performed to validate selected deregulations in gene expression after inhibitor treatment (EC_50_, 16 h) in cell lines of different GCT subtypes (SE: TCam-2, CC: JAR, YST: GCT72) (Fig. 4B). In the tested cell lines, all selected genes (Quisinostat: *DHRS2*; JIB-04: *DDIT4*; Chaetocin: *CD36*; MZ-1: *EIF3CL*; LP99: *SAMD3*; PRT4165: *ACE2*; GSK343: *FOS*) showed similar deregulations in gene expression as found in the RNA seq analysis .

### *Epi-drug treatment diminishes proliferation *via* cAMP, JAK-STAT and WNT signaling pathways*

A proteome profiler phospho-kinase array was used to detect phosphorylation of well-known kinase targets (Fig. 4C, Additional file 9: Fig. S9A–D). CREB, STAT3 and STAT5 phosphorylation and total levels of β-Catenin and HSP60 were commonly decreased upon treatment with Quisinostat, JIB-04, Chaetocin, MZ-1, LP99, PRT4165, GSK343 for 16 h in 2102EP. ERK1/2 phosphorylation was also decreased upon JIB-04, MZ-1, LP99, PRT4165 and GSK343 treatment, while p53 phosphorylation at serine (S) 64 was commonly increased, with the exception of Quisinostat and GSK343. Levels of p53 S15 phosphorylation were increased upon JIB-04 and Chaetocin treatment, but decreased when using the other epi-drugs (except for Quisinostat showing no alteration). We detected a broad variety of changes in phosphorylation after application of the BRD reader targeting drugs MZ-1 and LP99, as well as in PRT4165 and GSK343 treated cells, like Src, WNK1, Yes and RSK1/2 (Fig. 4C). Thus, epi-drug treatment affected activity of signaling pathways, resulting in altered phosphorylation states of related kinases.

### Potential co-treatment of GCTs with inhibitors against epi-drug induced target genes

**Fig. 5:**
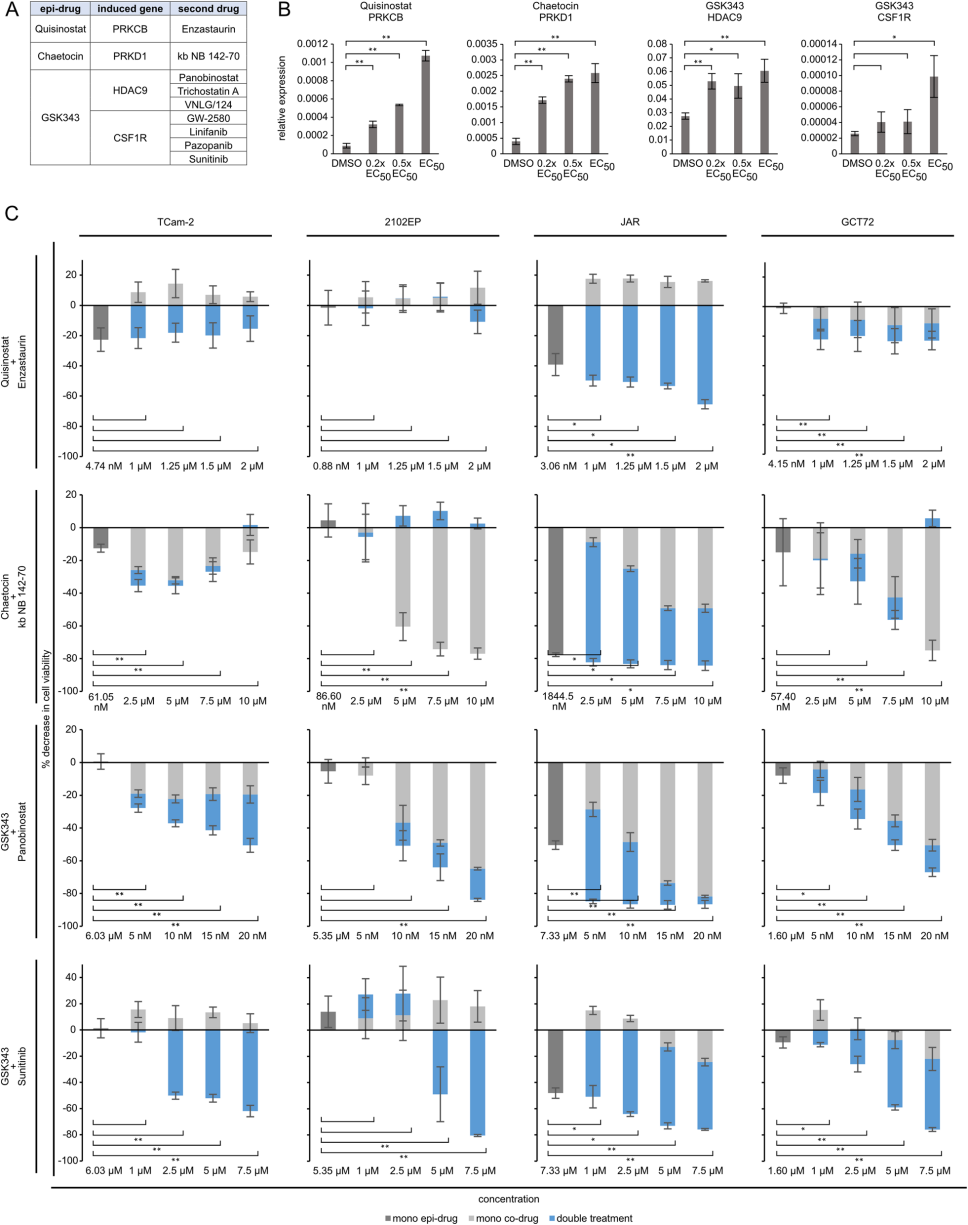
Potential targets for an epi-drug co-treatment. **A** Potential targets for co-treatment identified via PINA analysis of genes (> log_2_4) upregulated by an epi-drug treatment [51]. **B** qRT-PCR validation of upregulation of potential co-treatment target genes after low dose treatment with 0.2 × EC_50_ and 0.5 × EC_50_ and EC_50_ for comparison. Quisinostat: 0.2 × EC_50_: 0.35 nM, 0.5 × EC_50_: 0.88 nM, EC_50_: 1.76 nM; Chaetocin: 0.2 × EC_50_: 34.64 nM, 0.5 × EC_50_: 86.60 nM, EC_50_: 173.2 nM; GSK343: 0.2 × EC_50_: 2.14 µM, 0.5 × EC_50_: 5.36 µM; EC_50_: 10.71 µM). Asterisks indicate significant changes between treatment and solvent control (*p < 0.05, **p < 0.005). **C** Decrease in cell viability of mono versus combination treatment of indicated drugs . Cells were pretreated with the 0.5 × EC_50_ concentration of each epi-drug for 24 h, followed by treatment with different concentrations of the co-drug for 24 h and 48 h. Differences in cell viability are given in relation to the solvent controls. Raw data is given in Additional file 10: Fig. S10A. Asterisks indicate significant changes between double and both mono-treatments (*p < 0.05, **p < 0.005)

We further asked, if genes strongly induced by each epi-drug might be suitable targets for a combinatory approach. Therefore, we screened the PINA database for drugs targeting these significantly deregulated genes (fold change > log_2_2) [51]. Quisinostat induced upregulation of *PRKCB,* which is targetable by Enzastaurin (currently in glioblastoma treatment phase 3 trial) (Fig. 5A). Chaetocin induced upregulation of *protein kinase D* (*PRKD1*), which is targetable by kb NB 142–70 (Fig. 5A). For GSK343 induced *HDAC9,* three inhibitors were found: Panobinostat, Trichostatin A and VNLG/124 (Fig. 5A). GW-2580, Linifanib (therapy of hepatocellular carcinoma, phase 3 trial; other solid cancers, phase 2 trial), Pazopanib and Sunitinib were suitable inhibitors for the tyrosine-protein-kinase *CSF1R* (Fig. 5A). In summary, these drugs could be potential candidates for an efficient combination therapy with related epi-drugs (Fig. 5A). We assessed whether lower doses of the epi-drugs were able to induce upregulation of the afore-mentioned genes (Fig. 5B). Indeed, after treatment with corresponding 0.2 × EC_50_, 0.5 × EC_50_ and EC_50_ concentrations of the epi-drugs (Quisinostat: 0.35, 0.88, 1.76 nM, Chaetocin: 34.64, 86.60, 173.20 nM, GSK343: 2.14, 5.36, 10.71 µM), upregulation of *PRKCB* (Quisinostat), *PRKD1* (Chaetocin), *HDAC9* and *CSF1R* (GSK343) was observed (Fig. 5B). Next, we performed XTT assays in GCT cell lines using the epi-drugs alone and in combination with the identified co-drugs in varying concentrations, demonstrating that co-application considerably reduced viability of GCT cells compared to the mono-application and the solvent controls (Fig. 5C, Additional file 10: Fig. S10A).

The combination of Quisinostat and Enzastaurin as well as Chaetocin and kb NB 142–70 significantly reduced cell viability in JAR (30% and 5%) and GCT72 (10% and 10%) cells compared to the mono treatments, while having little to no effect on TCam-2 and 2102EP cells. The two combinations (Panobinostat and Sunitinib) with the PRC2i GSK343 showed promising results in all cell lines, significantly reducing cell viability compared to the mono treatments of the drugs. The combinatorial effect for GSK343 was strongest in TCam-2 (30%) and 2102EP (13%) upon treatment with 20 nM Panobinostat. The combination of GSK343 with Sunitinib was also effective in these two cell lines, since the mono treatments did not reduce cell viability. he combination led to a reduction of cell viability by 60% (TCam-2) and 80% (2102EP).

## Discussion

**Fig. 6:**
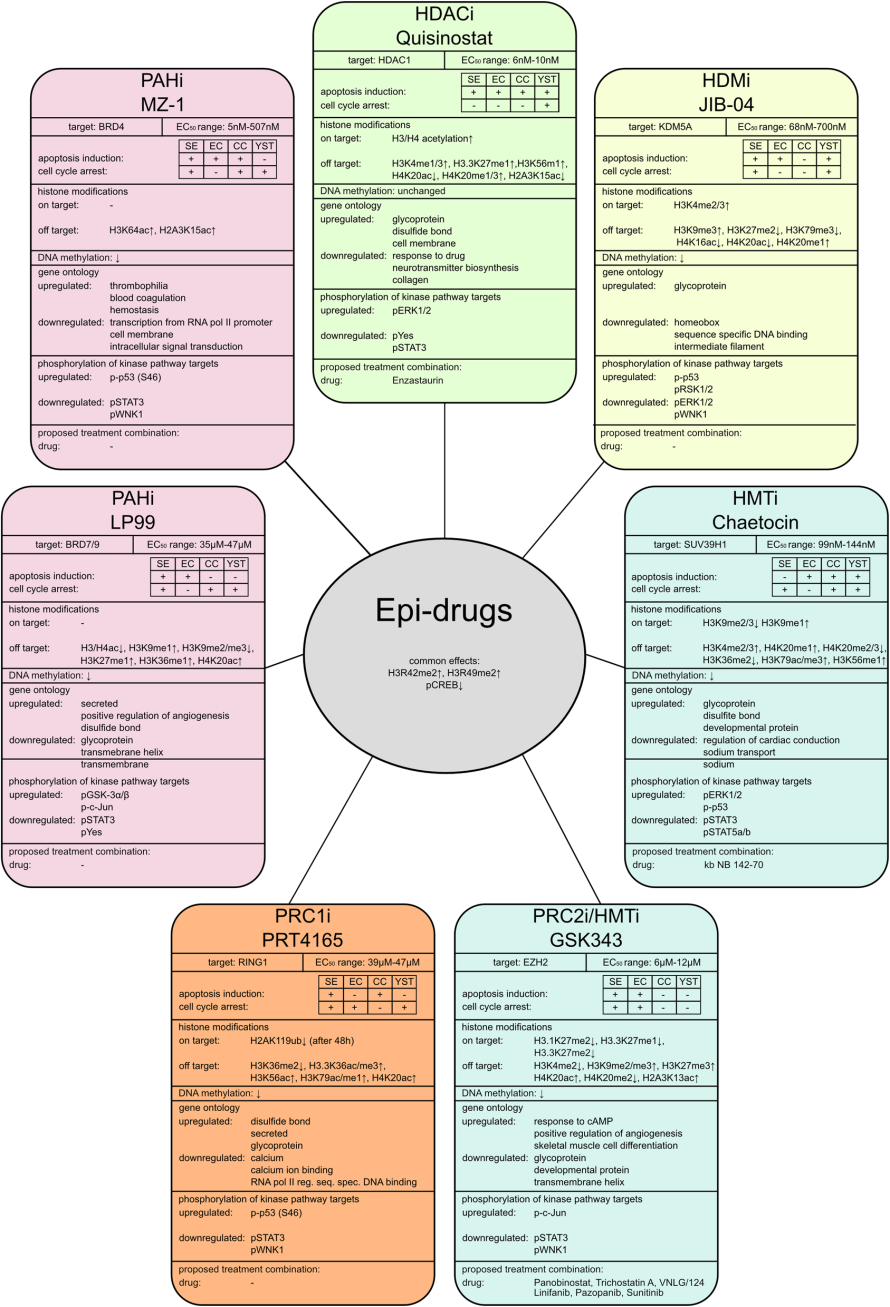
Summary of the epi-drug effects found in this study. EC_50_: half-maximal effective concentration where the cell viability has 50% compared to the solvent control. HDACi: histone deacetylase inhibitor, HMTi: histone methyltransferase inhibitor, HDMi: histone demethylase inhibitor, PAHi: acetylated histone binding protein inhibitor, PRCi: polycomb-repressive complex inhibitor, ↑: upregulation, ↓: downregulation

In this study, 16 different and selective epi-drugs were analyzed for their potential to treat (cisplatin-resistant) GCTs. Of these, seven were selected for further analyses due to their cytotoxicity at low nano- (Quisinostat, JIB-04, Chaetocin and MZ-1) to micromolar (LP99, PRT4165, GSK343) concentrations (Fig. 6). Most of the analyzed epi-drugs induced either apoptosis or cell cycle arrest in a GCT cell line-dependent manner (Fig. 6). In contrast, fibroblasts responded with no cell cycle arrest and only minor apoptosis induction. Additionally, other urological malignancies, like UC, PCa and RCC, were also sensitive towards the selected epi-drugs, since first cytotoxicity screens showed promising results for these entities. However, more research using different cell lines representing various subtypes of each entity is necessary. Thus, due to the focus of this study on GCTs, epi-drugs provide a potential therapeutic option for the treatment of SE, EC, and YST, as well as their cisplatin-resistant sublines, as they are more sensitive toward these epigenetic inhibitors compared to non-cancerous fibroblasts.

Regarding the cytotoxic efficacy of the HDACi Quisinostat, targeting HDAC1/2/4/10/11 [23, 52–54], we found a comparable cytotoxicity as previously observed with Panobinostat in EC cell lines, while it showed higher efficacy compared to the pan-HDACi Belinostat or Vorinostat/SAHA. Nevertheless, compared to the HDAC1/2-specific HDACi Romidepsin [16, 55], GCT cell lines responded less sensitive to Quisinostat treatment. However, Quisinostat has an improved pharmacokinetic profile compared to Romidepsin [56]. Some of the described epi-drugs have already been tested *in vivo* using mouse xenograft models, i.e. JIB-04 has been successfully tested in xenografts derived from lung cancer and Chaetocin in hepatoma and ovarian cancer xenografts [35, 57, 58]. The second-generation HDACi Quisinostat has already been included in clinical phase 2 trials as single treatment of T-cell lymphomas and as a second line treatment in combination with Paclitaxel and Carboplatin in ovarian cancer (ClinicalTrials.gov identifier NCT01486277 and NCT02948075), providing good requirements for clinical use of these epi-drugs.

We identified four druggable genes (*CSF1R*, *HDAC9*, *PRKCB*, *PRKD1*) upregulated by the epi-drugs (Chaetocin, GSK343, Quisinostat). Thus, drugs targeting these genes are putative combination partners for co-application with the corresponding epi-drug. Indeed, our analyses demonstrated that a combinatory application (Quisinostat + Enzastaurin; Chaetocin + kb NB 142-70; GSK343 + Panobinostat/Sunitinib) reduced the viability considerably compared to mono application in a cell line-dependent manner, suggesting that these drugs are promising combination partners for related epi-drugs. Nevertheless, the suitability as a therapeutic option for GCTs and other urological malignancies needs to be extensively analyzed in future studies.

We also characterized the molecular and epigenetic effects of each tested epi-drug. Beyond the expected histone modifications, mass spectrometry analyses revealed many off-target effects on the epigenetic landscape. For example, MZ-1, a reader of the epigenetic code, induced H3K64ac and H2A3K15ac, which are both modifications with rather unknown regulation and function, while PRT4165, an ubiquitin ligase RING1 inhibitor, induced various histone methylation and acetylation changes (e.g. H3K36ac/me2/me3, H3K79ac/me1). Regarding H2AK119ub after PRT4165 treatment , Ismail et al. [50] observed diminished H2AK119ub within one hour of treatment. Even though similar observations have been found in another study using 92.1 uveal melanoma cells [59], reduced H2AK119ub levels could not be seen in MP38 cells, which displayed low basal activity of PRC1. Additionally, Desai et al. observed a decrease in H2AK119ub upon treatment with PRT4165 for up to 36 h in undifferentiated KIND-1 (embryonic stem) cells, which displayed high RING1 protein levels, while BMI1 protein levels were rather low [60]. Our re-analysis of microarray data (Additional file 1: Fig. S1A, D) demonstrated that 2102EP cells and EC tissues also showed high *RING1* and low *BM1* expression. Hence, the authors conclude that due to the low *BMI1* expression in 2102EP, the decrease in H2AK119ub after PRT4165 treatment is detectable  at time-points later than 24 h (Additional file 6: Fig. S6E).

Most of the tested epi-drugs in this study were less sensitive in CC cell lines. CC is not only a cancer of the testis, but is also one of the most aggressive gestational trophoblastic neoplasia due to its propensity to invade the vascular system [61]. Specifically, CC consists of cytotrophoblast and syncytiotrophoblast cells [62, 63]. A possible reason for their intrinsic potential to evade cytotoxicity from epi-drugs could be explained by the presence of CC stem cell-like cells, i.e. trophoblast stem-like cells. The existence of a subpopulation representing early trophoblasts with trophoectoderm stem cell-like characteristics has been reported in CC [64, 65]. Moreover, Stichelbout et al. emphasized SALL4 being a useful marker to distinguish CC from placental site/epithelioid trophoblastic tumors [66]. More recently, Peng et al. hypothesized that the CC stem cell-like cell fate is influenced by *miR-497-5p* and its target gene *SALL4*. Specifically, DNA hypermethylation of the *miR-497-5p* coding sequence resulted in silencing of its expression, eventually leading to enhanced *SALL4* transcription in the CC cell line JEG-3 [67]. Subsequently, decreased sensitivity toward methotrexate, 5-fluoruracil, dactinomycin and etoposide was observed in *SALL4*-overexpressing JEG-3 CC cells [67]. Though, a CC stem cell-like subpopulation could be an explanation for CC being generally less affected by most tested epi-drugs, further investigations are needed to unravel this intrinsic drug resistance.

**Fig. 7:**
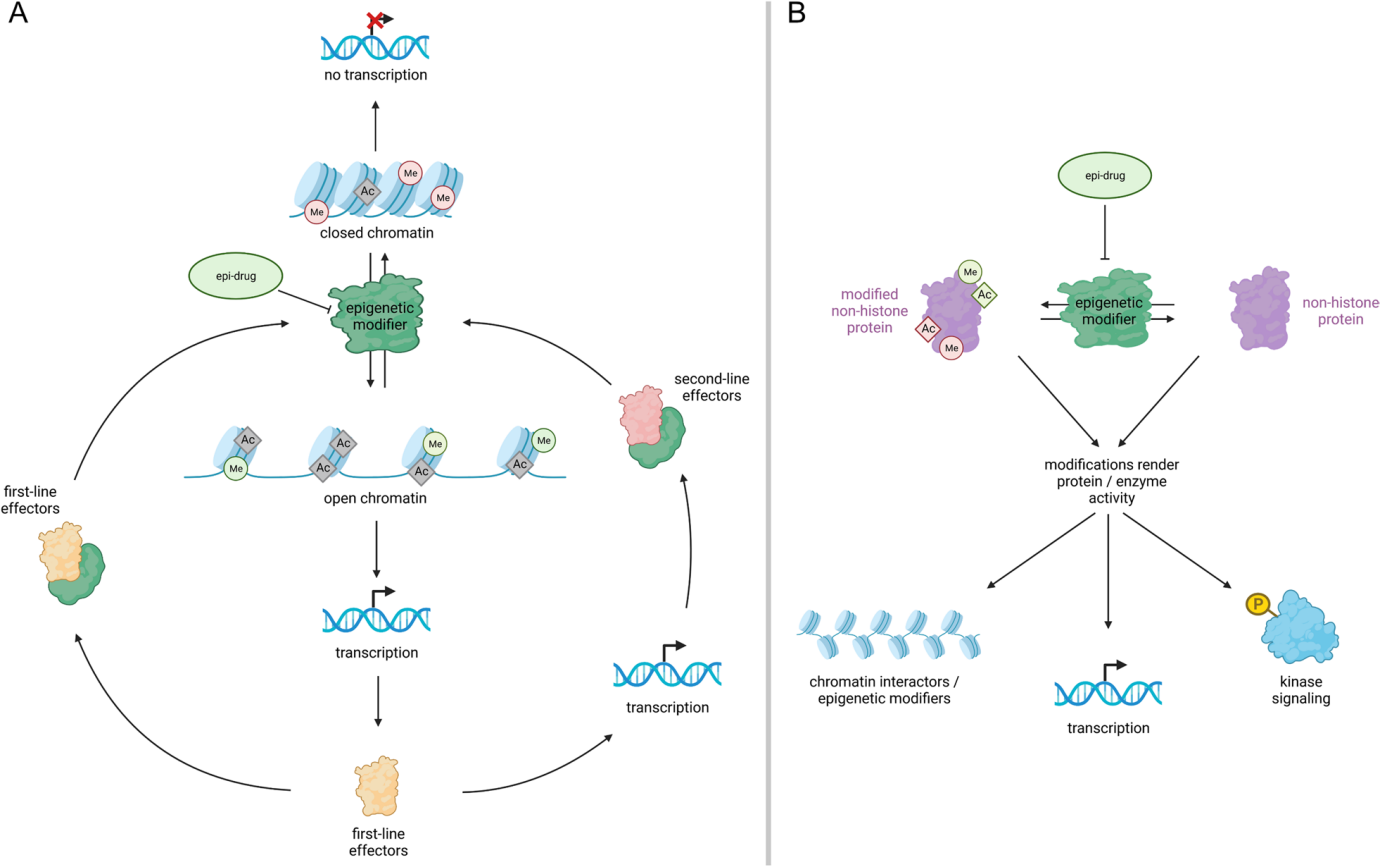
Modes of action of epi-drugs explaining the manifestation of off-target effects. **A** Epi-drug treatment leads either to closed chromatin silencing transcription or to open chromatin facilitating transcription of transcriptional regulators and signaling molecules (first-line effectors). These first-line effectors can directly influence the activity of epigenetic modifiers (left side) or induce expression of additional factors known to affect epigenetic modifiers (second-line effectors, right side). **B** Epigenetic modifiers may regulate the activity of non-histone proteins through post-translational modification. These proteins include chromatin interactors, transcription factors and kinases and may in turn affect chromatin accessibility, transcription and signaling pathways. Created with bioRender.com

How can we explain the detected off-target effects on the epigenetic landscape? Expression of alternative target molecules of the same enzyme class as the primary target of each epi-drug is quite low in GCT tissues and cell lines (Additional file 10: Fig. S10B, C), arguing for a high specificity of used epi-drugs. Nevertheless, we cannot fully rule out inhibitor target promiscuity as a reason for off-target effects. We hypothesize that each epi-drug induces changes in the histone modification landscape that influence expression of transcriptional regulators and signaling molecules (first-line effectors) (Fig. 7A). These first-line effectors either influence activity of epigenetic modifiers directly, or induce expression of additional factors known to influence epigenetic modifiers (second-line effectors) (Fig. 7A).

Of note, H3R49me2 was commonly found to be increased upon all tested epi-drug treatments. Unfortunately, there is a lack of studies investigating the function of this modification. The arginine residue 49 is thought to interact with the DNA and postulated to regulate gene expression by affecting H3K56ac (transcriptional activation) [68–70]. To a lesser extent, H3R42me2, linked to transcriptional activation, was increased under all treatments [71].

In addition to the changes in histone modifications, epi-drug treatment (except HDACi Quisinostat) affected the DNA-methylation status, i.e. decreased 5mC. Passive DNA-demethylation occurs over several cycles of replication; therefore, the 5mC decrease after 24 h is more likely due to active DNA demethylation (ADD). We observed a tendency for  decreased 5mC, which was accompanied by 5hmC accumulation. 5hmC formation is the first step of ADD and mediated by the TET enzymes (TET1—3) [72]. Thus, treatment of epi-drugs influences the DNA methylation landscape, i.e. leads to a decrease in 5mC and an increase in 5hmC, which is indicative of an ADD process. It needs to be mentioned that we analyzed quite early time points after epi-drug application (24 h), since at later time points levels of apoptosis were too high to guarantee reliable measurements. The analyzed time point might be too short to allow for genome wide changes in the DNA methylation landscape and might only detect effects caused by de novo ADD. Therapeutic options using DNA methyltransferase (DNMT) inhibitors have been previously investigated in GCTs. As such, treatment with the DNA hypomethylating agent 5-azacitidine (5-aza) or a novel synthetic flavonoid compound (MLo1302) resulted in apoptosis induction, followed by a cell cycle disruption in EC cell lines and their cisplatin-resistant sublines [73, 74].

Further analyses are necessary to validate ADD via the oxidation pathway, which is preferentially utilized in GCTs [10].

Quisinostat treatment caused deregulations in expression of genes associated with ion-transport and -binding factors. Downregulation of transporters regulating the ion uptake and upregulation of channels mediating gradient-dependent flow of potassium and chloride ions have been shown to be pro-apoptotic via inducing water loss of the cells [75]. In turn, uptake of calcium and sodium ions is considered pro-apoptotic [76]. Quisinostat treatment led to an upregulation of the calcium channel subunits (*CACNA1A/B/C/G;* Additional file 11: Table S1 B), which is in line with Ca^2+^ uptake through calcium channels being linked to membrane depolarization and cell death in adrenal chromaffin cells [77]. In addition, several potassium channel subunits were upregulated after Quisinostat treatment (*KCN* genes), which could also result in pro-apoptotic release of potassium ions. Chaetocin treated cells showed downregulation of potassium and sodium transporters (*ATP1A2*), eventually leading to a reduction of K^+^ and accumulation of Na^+^, which have been associated with apoptosis in lymphoma cells [78].

JIB-04 and MZ-1 did not induce significant changes in the transcriptome of 2102EP. Nevertheless, one gene found significantly altered upon JIB-04 treatment (*DDIT4*,DNA damage-inducible transcript 4 protein) was also upregulated in H358 (non-small cell lung cancer cells) [35]. Another study found a decrease in expression of* HOX *genes, also showing a tendency towards downregulation in our study (Additional file 11: Table S1 D) [79]. The previously mentioned studies analyzed effects provoked by epi-drugs after 24 h, whereas our studies focused on effects after 16 h as cell viability decreased strongly at later time points. We therefore propose that transcriptional changes observed by different studies occur at later timepoints and depend on the cell line analyzed.

Phosphorylation of CREB decreased commonly upon treatment with the epi-drugs, suggesting reduced activity of several pathways like cAMP, MAPK, cGMP/PKG and PI3K/Akt. Reduced CREB phosphorylation has been associated with diminished proliferation and apoptosis rates in smooth muscle and neuronal cells [80, 81]. However, our functional annotation analyses revealed only upregulation of ‘cAMP response’ and ‘MAPK signaling pathway’ upon Quisinostat and GSK343 treatment. Although deregulations in expression of signaling pathway molecules were not prominent, activity of these pathways could also be mediated through the epigenetic modifiers themselves, by modifying transcription factors like p53 and STAT3 [82, 83]. These non-histone interactions are less studied than the histone modifications but might still play a role in the mode of action of epi-drugs and should be paid more attention. Thus, we provide an alternative hypothesis involving non-histone interactions to explain epigenetic off-target effects (Fig. 7B). Here, we propose that the tested epi-drugs influence post-translational modifications of proteins, like kinases and transcription factors (e.g. by acetylation), thereby rendering their activity, which in turn might influence the epigenetic landscape (Fig. 7B) [84–86]. In support of our theory, it has been shown that epigenetic modifiers can target chromatin interactors like RAG2 and methyltransferases KMT5A (alternative name SET8, monomethylation of H4K20 [87]) and DOT1L (methylation of H3K79 [88]) and regulate their activity or target binding properties, thereby providing a further mechanism for indirect mediation of chromatin accessibility [89].

### Summary and conclusion

This study demonstrated that a variety of epigenetic modifiers can successfully be targeted as a treatment option for urologic malignancies (GCT, UC, RCC, PCa), thereby inducing apoptosis and/or cell cycle arrest. Cell lines from different pan-urologic cancer entities were highly sensitive toward the epi-drugs within a therapeutic window, mostly ruling out effects on healthy cells, e.g. fibroblasts. Additionally, we provide a detailed analysis of drug related (off-target) effects on the histone modification landscape. Further, we gained mechanistical insights by characterizing each epi-drug on transcriptional level and analyzing related signaling pathways. Our data provide a valuable source of information on tested epi-drugs, which might be of benefit for drug combination attempts and patient/therapy risk assessment.

## Material and methods

### Cell culture

All GCT cell lines were grown as described previously [90, 91]. Cell lines and corresponding media used are listed in the supplement (Additional file 12: Table S2A) Briefly, GCT and fibroblast cell lines were grown at 37 °C and 7.5% CO_2_, while bladder, prostate and renal carcinoma cell lines were grown at 37 °C and 5% CO_2_. Short tandem repeat (STR) profiles of all cell lines are checked on a regular basis and are available upon reasonable request. All cell lines are scrutinized for mycoplasma contamination on a regular basis using PCR strategy. Briefly, medium from cultured cells was boiled at 95 °C for 5 minutes (min) and 2 µl used in the PCR. The primers were used as a mix of ten primers. Sequences are given in Additional file 12: Table S2B.

### Cell viability assay

The triphenyl tetrazolium chloride (XTT) assays were performed as described previously [90]. In summary, for GCT and fibroblast cell lines 5 × 10^3^ cells (3 × 10^3^ for UC, PCa and RCC cell lines) per 96-well were seeded and different drug concentrations (see Additional file 2: Fig. S2, Additional file 3: Fig. S3) or corresponding solvents were added to the cells the following day. For drug details see Additional file 12: Table S2 C. Each condition was analyzed in four technical replicates and EC_50_ values were calculated by GraphPad Prism v8.

### Flow cytometry

Flow cytometry analyses of apoptosis rates and cell cycle distribution was performed by Annexin V/propidium iodide (PI) or PI staining, respectively, using MACSQuant Analyzer 10 (Miltenyi Biotech, Bergisch Gladbach, Germany) after 16 h of treatment with the corresponding EC_50_ concentrations for each cell line. For Annexin V/PI staining, cells were washed with Annexin V Binding Buffer before being resuspended in 70 µl Annexin V Binding Buffer and 2.5 μl Annexin V FITC-conjugate (both Miltenyi Biotec, Bergisch Gladbach, Germany) and 15 µg PI (Sigma-Aldrich, Taufkirchen, Germany) and incubated in the dark at room temperature for 15 min. 500 µl of Annexin V Binding Buffer was added before measurement. For the cell cycle analysis, cells were washed with PBS (phosphate buffered saline), fixed by ice cold 70% ethanol and stained with 2 µg/ml PI and 200 µg/ml RNaseA (Quiagen, Hilden, Germany 100 mg/ml) in PBS shortly before every measurement. At least 5 × 10^4^ cells were counted and analyzed using the MACSQuant software v 2.13.0.

### DNA, RNA, protein and histone isolation

DNA was isolated using the QIAamp DNA Mini Kit (Quiagen, Hilden, Germany) according to the manual. RNA from cell lines was isolated using the RNAeasy Mini Kit (Qiagen, Hilden, Germany) according to the manual. Proteins were extracted using RIPA buffer (Cell Signaling, Frankfurt am Main, Germany) containing protease inhibitor tablets (Roche, Basel, Switzerland). Histones were extracted using the Histone Extraction Kit according to the manufacturer protocol (Abcam, Cambridge, UK). Protein concentrations were assessed by the BCA Protein Assay Reagent Kit (Thermo Fisher Scientific, Schwerte, Germany). RNA concentrations as well as 260/280 nm, 260/230 nm purity ratios were determined by NanoDrop measurement (Thermo Fisher Scientific, Schwerte, Germany).

### DNA slot blot

Purity of DNA was verified using NanoDrop 260/280 ratio and agarose gel electrophoresis. DNA was diluted in H_2_O to 200 ng, 100 ng and 50 ng in 200 µl per slot. Slot-Blotter device (Carl Roth GmbH + Co KG, Karlsruhe, Germany) was assembled as according to the manual. DNA was blotted onto a positively charged nylon membrane (Carl Roth GmbH + Co KG, Karlsruhe, Germany) using vacuum application. Three identical membranes were prepared. The membranes were air-dried for 15—20 min and UV-crosslinked (20 s (s), 1200 mJ/cm^2^). One membrane was stained with methylene blue (0.02% methylene blue (Merck Millipore, Darmstadt, Germany) in 0.3 M sodium acetate (pH 5.2)) to validate DNA loading across the samples. The remaining two membranes were blocked in 5% milk powder in PBST for 1 h at RT. Membranes were incubated with 5mC or 5hmC antibody overnight at 4 °C. After washing, membranes were incubated with secondary antibody for 2 h at RT. Signals were detected using the ChemiDoc Imaging Systems (Bio-Rad Laboratories, Feldkirchen, Germany). See Additional file 12: Table S2 D for antibody details.

### Western blot

Western Blots were performed as described previously [90]. In total, 20 µg of whole cell protein lysates or 3 µg of histone extract were used for western blotting. β-Actin was used as housekeeper and loading control for whole cell lysates. Western blots of histone extracts were stained with Coomassie Brilliant Blue R 250 (Roth, Karlsruhe, Germany) and total H3 or H4 antibody to verify equal loading. For antibody details, see supplement (Additional file 12: Table S2 D). The ChemiDoc Imaging System (Bio-Rad Laboratories, Feldkirchen, Germany) was used for detection. Pixel density was analyzed using the ‘Image Lab’ software provided by Bio-Rad.

### Kinase signaling array

For the detection of phosphorylated kinase targets, the Proteome Profiler Human Phospho-Kinase Array Kit (R&D Systems, Abingdon, UK) was used according to the manufacturer’s instructions. The ChemiDoc Imaging System (Bio-Rad Laboratories, Feldkirchen, Germany) was used for detection. 2102EP cells were harvested after 16 h of treatment with the epi-drugs. Pixel density was analyzed using Fiji ImageJ 1.53c [92].

### Quantitative RT-PCR

Quantitative RT-PCR (qRT-PCR) was performed as described previously [93]. A total amount of 1 µg of RNA was in-vitro transcribed into cDNA by using Oligo(dT)18 Primer and Maxima H Minus Reverse Transcriptase (200 U/µL) (all Thermo Fisher Scientific). qRT-PCR runs were performed on a 384-well C1000 cycler (BioRad, Feldkirchen, Germany). All samples were analyzed in technical triplicates using 7.34 ng of cDNA for each replicate and the SYBR-green-based Luna Universal qPCR Master Mix (New England Biolabs, Frankfurt am Main, Germany). At the end of each run, melting curve analyses were performed. Oligonucleotide sequences are given in the supplement (Additional file 12: Table S2 B).

### Analysis of histone modifications via mass spectrometry

2102EP cells were treated with the EC_50_ concentrations for Quisinostat, JIB-04, Chaetocin, MZ-1, LP99, PRT4165, GSK343 and DMSO as solvent control. The cells were harvested via trypsinization after 16 h. The mass spectrometric analysis of histone modifications was outsourced to the Mod Spec service by Active Motif (Carlsbad, CA, USA). Histone extraction and mass spectrometric analysis was performed as described previously [94]. Raw data were transformed into z-scores as the input. The heatmap was generated in R using ‘pheatmap’ with default settings and provided by Active Motif.

### RNA sequencing

Transcriptome analysis has been performed as previously described at the core facility ‘Genomics & Transcriptomics’ (Prof. Dr. Karl Köhrer, Biomedical Research Center, Heinrich Heine University, Düsseldorf, Germany) and has been publicly shared via GEO (https://www.ncbi.nlm.nih.gov/geo/, GSE189472) [90, 95]. RNA samples were quantified and measured as previously described [90, 95]. Statistical analyses were performed using the ‘Differential Expression in two groups’ tool (version 1.02) (Qiagen) and the resulting *p* values were corrected for multiple testing by false discovery rate (FDR). Accompanying PCA and heatmap were generated using Qlucore Omics Explorer 3.7 (Qlucore AB, Lund, Sweden). Values of solvent controls wereaveraged from the studies GSE189472, GSE190792 and GSE190022 [95].

### Re-analysis of microarray data of GCT tissues and cell lines

Experimental setup and microarray data of GCT tissues (Affymetrix expression array) and cell lines (Illumina HT-12v4 expression microarray) have already been published [10, 15, 16, 19–22, 90, 96]. The microarray data were re-analyzed in context of this study and includes normal testis tissue (*n* = 4), GCNIS (*n* = 3), SE (*n* = 4), EC (*n* = 3), TE (*n* = 3) and mixed non-seminomas (*n* = 4). All tumors were classified according to the WHO classification of tumors based on their histology and assessment of tumor or GCNIS amount. GCT cell line datasets included TCam-2 (*n* = 5), 2102EP (*n* = 5), NCCIT (*n* = 4) and JAR (*n* = 2). Data are available via GEO (https://www.ncbi.nlm.nih.gov/geo/, GSE71239, GSE71269, GSE79065, GSE87477 and GSE60698). Expression intensities < 10 (Affymetrix) and < 8 (Illumina) were considered as not expressed based on expression intensities of *SOX2* and *SOX17* in SE (*SOX2*-, *SOX17* +) and EC (*SOX2* + , *SOX17*-) cell lines or tissues.

### Online tools and software

Analysis of RNA seq data was performed using the online tool DAVID Bioinformatics Resources 6.8 (https://david.ncifcrf.gov/) for functional annotations [97, 98]. Annotation databases used included UniProt Keywords, Gene Ontology biological process and molecular function and KEGG pathway. Interaction analysis was performed using the STRING algorithm (https://string-db.org) [99, 100]. The TCGA database was screened using cBioportal (https://www.cbioportal.org/) [44, 45]. The following cohorts were used for analysis: testicular germ cell tumors (149 samples, TCGA, PanCancer Atlas), urothelial carcinoma (413 samples, TCGA, Firehose Legacy), prostate adenocarcinoma (501 samples, TCGA, Firehose Legacy), kidney renal clear cell carcinoma (538 samples, TCGA, Firehose Legacy) and kidney renal papillary cell carcinoma (293 samples, TCGA, Firehose Legacy). Venn diagrams were created using interactivenn (http://www.interactivenn.net/) [101] and the Circos Table Viewer was used to generate circos diagrams (http://mkweb.bcgsc.ca/tableviewer/) [102]. The functional annotation graphics were designed using a word cloud tool (https://www.wordclouds.com/) by Zygomatic (Vianen, Netherlands). PINAv3.0 (https://omics.bjcancer.org/pina/) was used to identify drugs suitable to target molecules induced by epi-drugs [51]. Proposed mode of action figures werecreated with bioRender (https://app.biorender.com/) (Toronto, Ontario, Canada).

### Statistical analyses

A two-tailed Student’s *t* test after confirming equality of two variances according to the *F* test has been performed to analyze differences between groups (*p < 0.05, **p < 0.005).

## Supplementary Information

**Additional file 1: Figure S1**. Identification of 16 potential epigenetic targets for GCT therapy. **A** Re-analysis of gene expression of epigenetic modifiers in GCT tissue samples (microarray data Affymetrix, GCNIS (*n* = 3), SE (*n* = 4), EC (*n* = 3), TE (*n* = 3) and mixed non-seminomas (*n* = 4)). **B**, **C** A Venn diagramof commonly expressed epigenetic modifiers in GCT tissues (**B**) and cell lines (**C**) highlighted 80 commonly expressed genes in GCT tissues and 33 in GCT cell lines . **D** Re-analysis of the 80 commonly expressed epigenetic modifiers of GCT tissues in GCT cell lines (microarray data Illumina, TCam-2 (*n* = 5), 2102EP (*n* = 5), NCCIT (*n* = 4) and JAR (*n* = 2)). **E** Final list of 35 target genes (including BRD4 and EZH2) as well as corresponding drugs. **F** Western blot analysis verifying protein levels of BRD4, VHL and EZH2 in GCT cell lines. β-Actin was used as loading control. Expression microarray data were re-analyzed in context of this study [10, 15, 16, 19–22, 90, 96]. HDAC, histone deacetylase; HAT, histone acetyltransferase; HDM, histone demethylase; HMT, histone methyltransferase; PAH, proteins binding acetylated histones; PMH, proteins binding methylated histones; PRC, polycomb-repressive complex; ADD, active DNA demethylase; DNMT, DNA methyltransferase; PMDN, proteins binding methylated DNA; SE, seminoma; EC, embryonal carcinoma; YST, yolk-sac tumor; CC, choriocarcinoma.

**Additional file 2: Figure S2**. XTT data for 16 epigenetic inhibitors in three GCT cell lines and MPAF fibroblast cells (*n* = 4). Cells were treated once with indicated epi-drugs. Cell viability was detected after 24, 48, 72 and 96 h. The red line indicates 50% cell viability. Asterisks indicate significant changes between treatment and solvent control (after 48 h, time point chosen for EC_50_ calculation) (*p < 0.05, **p < 0.005).

**Additional file 3: Figure S3**. XTT data for eight epigenetic inhibitors in 14 GCT cell lines, fibroblast cells MPAF and HVHF2, and Sertoli cells FS1 (*n* = 4). Cells were treated once with indicated epi-drugs. Cell viability was detected after 24, 48, 72 and 96 h. The red line indicates 50% cell viability. Asterisks indicate significant changes between treatment and solvent control (after 48 h, time point chosen for EC_50_ calculation) (*p < 0.05, **p < 0.005).

**Additional file 4: Figure S4**. Epi-drugs decrease cell viability in GCTs and other urological entities. **A** EC_50_ values for each GCT cell line, fibroblasts and Sertoli cells and epi-drug treatment were calculated from data shown in Fig. S3. **B** Analysis of the mutational and gene expression alteration spectrum of the top eight target genes using the cBioPortal tool on the 'Testicular Germ Cell Tumors' cohort of TCGA. **C**–**E** XTT data for Quisinostat, JIB-04 and Chaetocin treatment in three cell lines each for UC (**C**), PCa (**D**) and RCC (**E**). The red line indicates 50% cell viability. Asterisks indicate significant changes between treatment and solvent control (after 48 h, time point chosen for EC_50_ calculation) (*p < 0.05, **p < 0.005). **F** EC_50_ values from (**C**), (**D**) and (**E**) calculated using GraphPad Prism v8. EC_50_: half-maximal effective concentration where the cell viability has 50% compared to the solvent control. SE, seminoma; EC, embryonal carcinoma; YST, yolk-sac tumor; CC, choriocarcinoma; SC, Sertoli cells; UC, urothelial carcinoma; PCa, prostate cancer; RCC, renal cell carcinoma.

**Additional file 5: Figure S5**. Raw data of flow cytometry analysis of Annexin V/PI (apoptosis) or PI staining only (cell cycle) of indicated cell lines (columns) after 16 h of treatment with indicated epi-drugs (rows).

**Additional file 6: Figure S6**. Mass spectrometry analysis of histone modifications arranged by frequency. **A**–**C** Circos diagrams are illustrating the difference in peptide frequency of given histone modifications of treated 2102EP cells compared to the solvent control. Histone modifications with a general peptide frequency of 10–100% (**A**), 1–10% (**B**) and 0–1% (**C**) are depicted separately for better comparison. Only differences in modification abundancy > 1 (10–100%), > 0.1 (1–10%) or > 0.01 (0.1–1%) were considered. **D** Depiction of the information given by the circos diagram rings. **E** Western blot analysis of histone extracts from PRT4165 or solvent control DMSO treated 2102EP cells after 24 h and 48 h. Coomassie staining is shown as loading control. **F** Western blot analysis of selected histone modifications in GCT cell lines. Cells were treated with indicated epi-drugs for 16 h. Total H3/H4 was used as loading control. ↑: upregulation, ↓: downregulation.

**Additional file 7: Figure S7**. **A** Unsupervised clustering heatmap of RNA seq data (DMSO control *n* = 3, epi-drugs *n* = 1). 2102EP were treated with indicated epi-drugs for 16 h. **B** STRING analysis of upregulated genes in Quisinostat treated (16 h) 2102EP cells.

**Additional file 8: Figure S8.** STRING analysis of deregulated genes after 16 h of indicated epi-drug treatments in 2102EP cells (DMSO control *n* = 3, epi-drugs *n* = 1). **A** LP99 upregulated genes, **B** LP99 downregulated genes, **C** PRT4165 upregulated genes, **D** PRT4165 downregulated genes, **E** GSK343 upregulated genes, **F** GSK343 downregulated genes.

**Additional file 9: Figure S9**. **A** Proteome Profiler Human Phospho-Kinase Array raw data. 2102EP were treated with indicated epi-drugs for 16 h (*n* = 2). **B** Membrane layout of kinase array with corresponding spotted antibodies given in **C.** Detectable dots (marked in red) were used for quantification with ImageJ as shown in **D**. Gray bar indicates cut-off value of 2500.

**Additional file 10: Figure S10**. **A** XTT cell viability raw data of co-treatments. Cells were pretreated with the 0.5 × EC_50_ concentration of each epi-drug for 24 h, followed by treatment with different concentrations of the co-drug for 24 h and 48 h.** B**,** C** Epi-drug side target gene expression in GCT tissues (**B**) (normal testis tissue (*n* = 4), GCNIS (*n* = 3), SE (*n* = 4), EC (*n* = 3), TE (*n* = 3) and mixed non-seminomas (*n* = 4) and cell lines (**C**) (TCam-2 (*n* = 5), 2102EP (*n* = 5), NCCIT (*n* = 4) and JAR (*n* = 2). Expression microarray data were re-analyzed in context of this study [10, 15, 16, 19–22, 90, 96]. IC_50_ values from published data for Quisinostat [23], JIB-04 [35], Chaetocin [31], MZ-1 [103], LP99 [36], PRT4165 [27] and GSK343 [34]. GCT, germ cell tumors; GCNIS, germ cell neoplasia in situ; EC, embryonal carcinoma; IC_50_, half-maximal inhibitory concentration; HDAC, histone deacetylase; BET, bromodomain and extra-terminal motif; PRC, poly repressive complex.

**Additional file 11: Table S1**. **A** Raw data of mass spectrometry analysis of histone modifications given as peptide frequency (*n* = 3). **B** Corresponding bar charts to (**A**) scaled to histone peptide frequency of 100%, 10% and 1%. **C–I** RNA seq data given as fold change compared to solvent control (log_2_) as well as DAVID functional annotation analysis for **C** Quisinostat, **D** JIB-04, **E** Chaetocin, **F** MZ-1, **G** LP99, **H** PRT4165 and **I** GSK343.

**Additional file 12: Table S2**. **A** Studied cell lines including appropriate culture conditions. **B** Sequences of used oligonucleotides. **C** Epi-drug solvents and suppliers. **D** Used antibodies.

## Data Availability

The datasets supporting the conclusions of this article are available in the NCBI Gene Expression Omnibus (GEO) database repository, [GSE71239, GSE71269, GSE79065 and GSE60698, current study GSE189472; https://www.ncbi.nlm.nih.gov/geo/]. Mutational and RNA expression data of GCT, UC, PCa and RCC were retrieved from cBioportal, a publicly available database: https://www.cbioportal.org/. The Mod Spec dataset supporting the conclusions of this article is included within the article (and its Additional file 12: Table S2 A, B).

## Abbreviations

ADD: Active DNA demethylation
BET: Bromodomain and extra-terminal motif
BRD: Bromodomain containing protein
CC: Choriocarcinoma
DNMT: DNA methyltransferase
EC: Embryonal carcinoma
EC_50_: Half-maximal effective concentration
epi-drugs: Drugs targeting epigenetic interactors
GCNIS: Germ cell neoplasia in situ
GCT: Germ cell tumors
h: Hours
HAT: Histone transferase
HDAC: Histone deacetylase
HDM: Histone demethylase
HMT: Histone methyltransferase
i: Inhibitor
IC_50_: Half-maximal inhibitory concentration
min: Minutes
p: Phosphorylation
PAH: Proteins binding acetylated histones
PCa: Prostate cancer
PMDN: Proteins binding methylated DNA
PMH: Proteins binding methylated histones
PRC: Polycomb-repressive complex
PROTAC: Proteolysis targeting chimera
-R: Cisplatin resistant clone
RCC: Renal cell carcinoma
s: Seconds
SC: Sertoli cells
SE: Seminoma
TCGA: The Cancer Genome Atlas
TE: Teratoma
ub: Ubiquitination
UC: Urothelial carcinoma
YST: Yolk-sac tumor
