# Supplementary figures and images for: Therapeutical interference with the epigenetic landscape of germ cell tumors: a comparative drug study and new mechanistical insights
Source: Clin Epigenetics. 2022 Jan 7;14:5. doi: 10.1186/s13148-021-01223-1 (PMC8742467; doi:10.1186/s13148-021-01223-1)

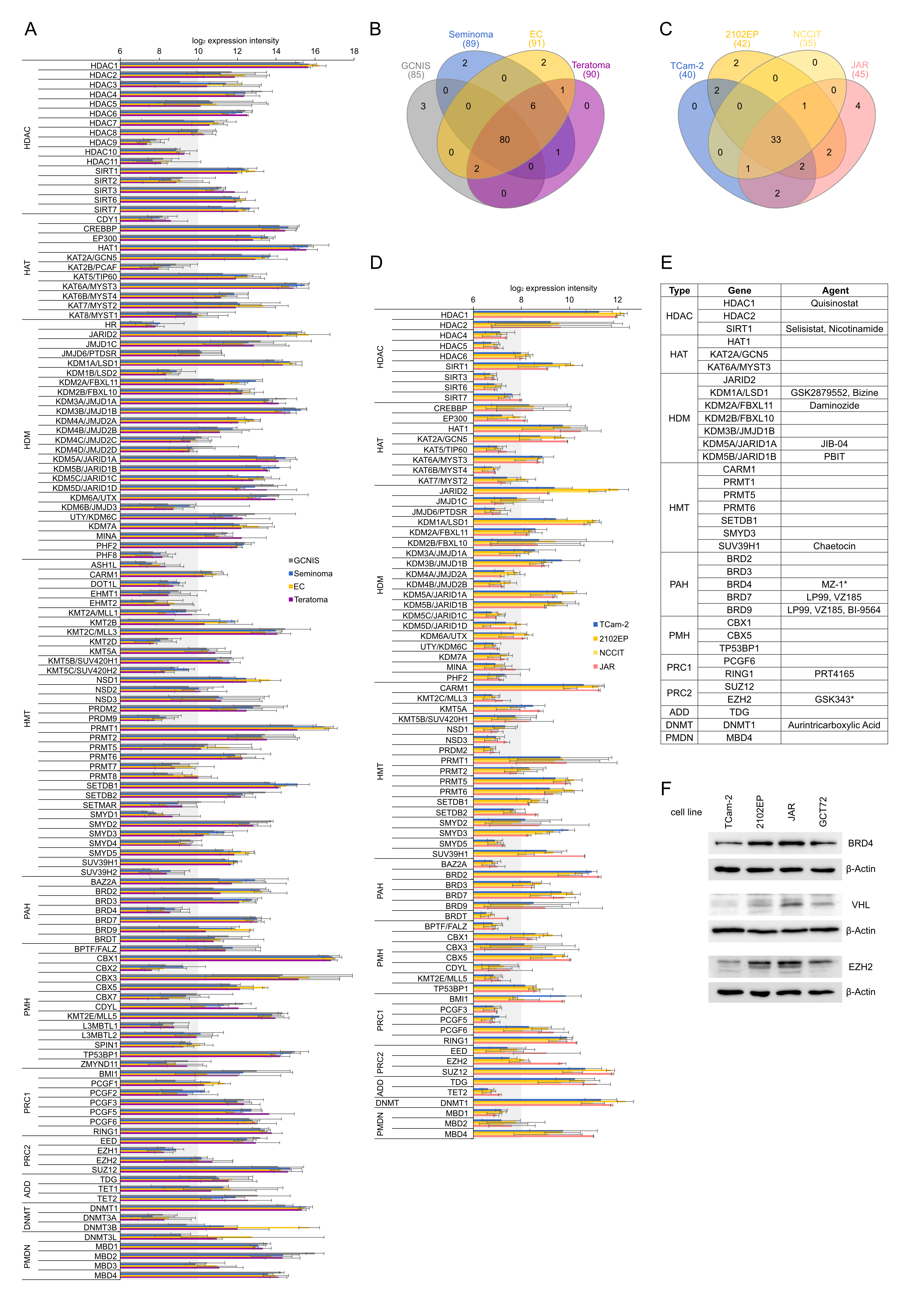

Supplement: Supplementary file 1 — Additional file 1: Figure S1. Identification of 16 potential epigenetic targets for GCT therapy. A Re-analysis of gene expression of epigenetic modifiers in GCT tissue samples (microarray data Affymetrix, GCNIS (n = 3), SE (n = 4), EC (n = 3), TE (n = 3) and mixed non-seminomas (n = 4)). B, C A Venn diagramof commonly expressed epigenetic modifiers in GCT tissues (B) and cell lines (C) highlighted 80 commonly expressed genes in GCT tissues and 33 in GCT cell lines . D Re-analysis of the 80 commonly expressed epigenetic modifiers of GCT tissues in GCT cell lines (microarray data Illumina, TCam-2 (n = 5), 2102EP (n = 5), NCCIT (n = 4) and JAR (n = 2)). E Final list of 35 target genes (including BRD4 and EZH2) as well as corresponding drugs. F Western blot analysis verifying protein levels of BRD4, VHL and EZH2 in GCT cell lines. β-Actin was used as loading control. Expression microarray data were re-analyzed in context of this study [10, 15, 16, 19–22, 90, 96]. HDAC, histone deacetylase; HAT, histone acetyltransferase; HDM, histone demethylase; HMT, histone methyltransferase; PAH, proteins binding acetylated histones; PMH, proteins binding methylated histones; PRC, polycomb-repressive complex; ADD, active DNA demethylase; DNMT, DNA methyltransferase; PMDN, proteins binding methylated DNA; SE, seminoma; EC, embryonal carcinoma; YST, yolk-sac tumor; CC, choriocarcinoma. [file 13148_2021_1223_MOESM1_ESM.tiff]

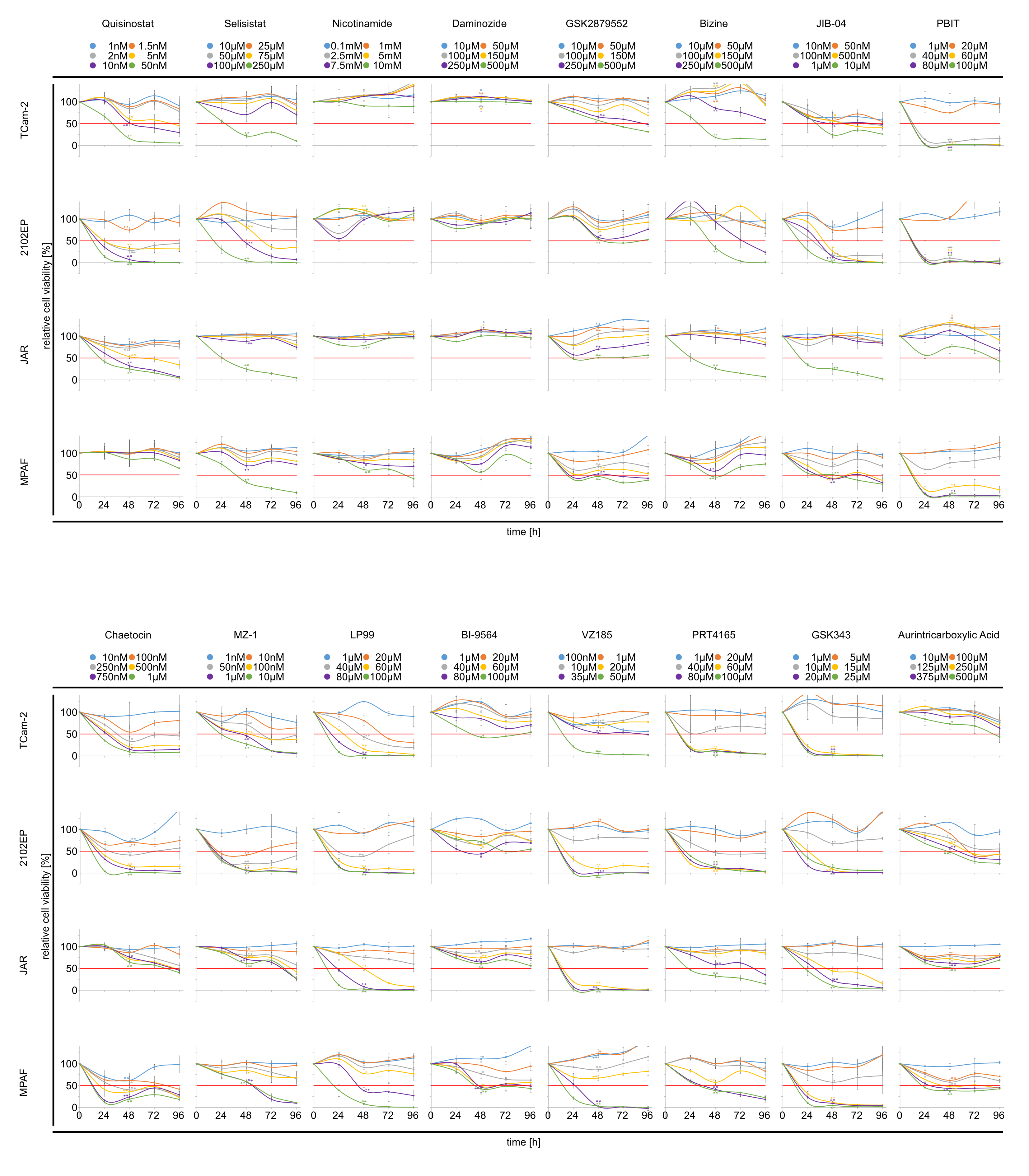

Supplement: Supplementary file 2 — Additional file 2: Figure S2. XTT data for 16 epigenetic inhibitors in three GCT cell lines and MPAF fibroblast cells (n = 4). Cells were treated once with indicated epi-drugs. Cell viability was detected after 24, 48, 72 and 96 h. The red line indicates 50% cell viability. Asterisks indicate significant changes between treatment and solvent control (after 48 h, time point chosen for EC50 calculation) (*p < 0.05, **p < 0.005). [file 13148_2021_1223_MOESM2_ESM.tiff]

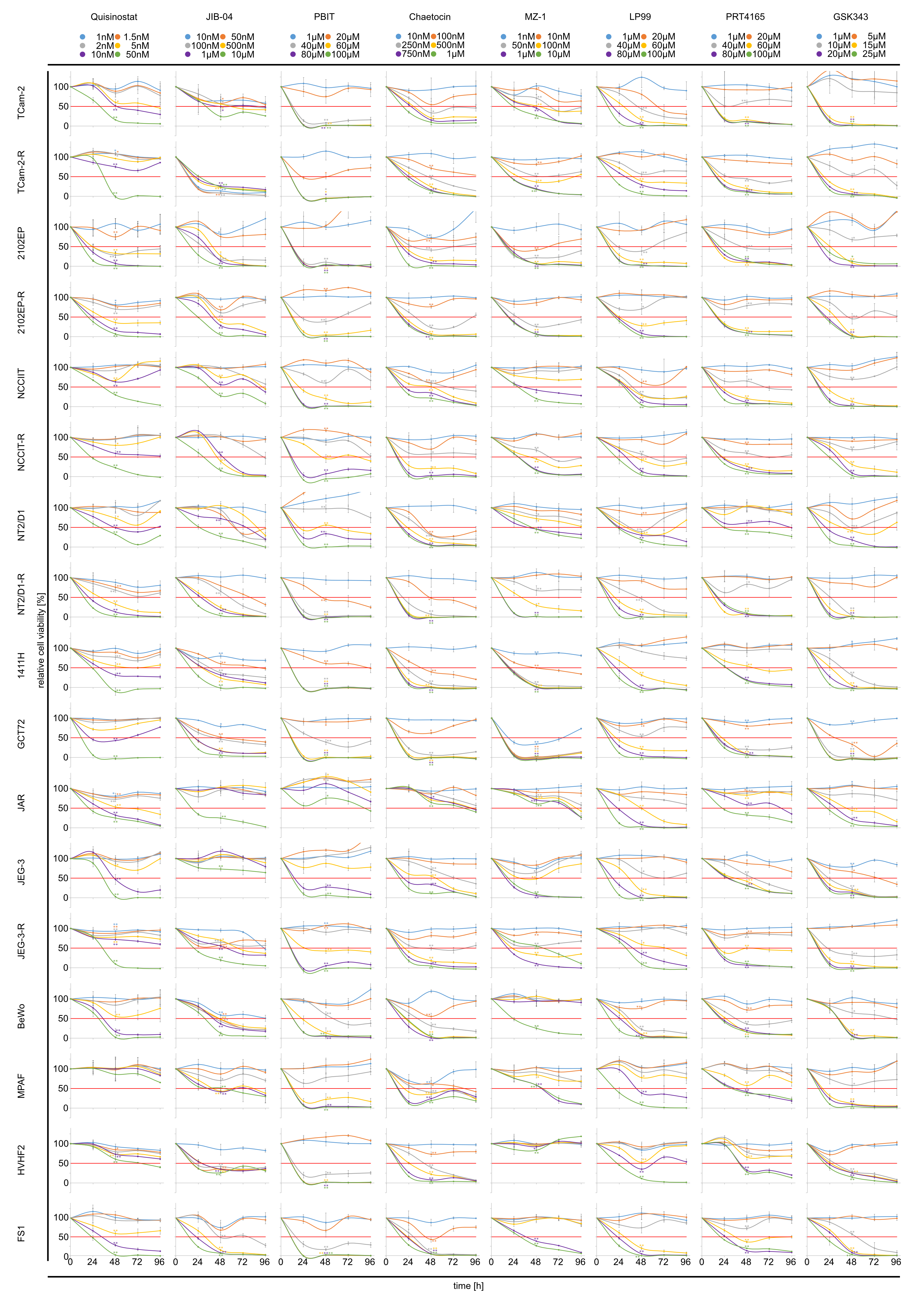

Supplement: Supplementary file 3 — Additional file 3: Figure S3. XTT data for eight epigenetic inhibitors in 14 GCT cell lines, fibroblast cells MPAF and HVHF2, and Sertoli cells FS1 (n = 4). Cells were treated once with indicated epi-drugs. Cell viability was detected after 24, 48, 72 and 96 h. The red line indicates 50% cell viability. Asterisks indicate significant changes between treatment and solvent control (after 48 h, time point chosen for EC50 calculation) (*p < 0.05, **p < 0.005). [file 13148_2021_1223_MOESM3_ESM.tiff]

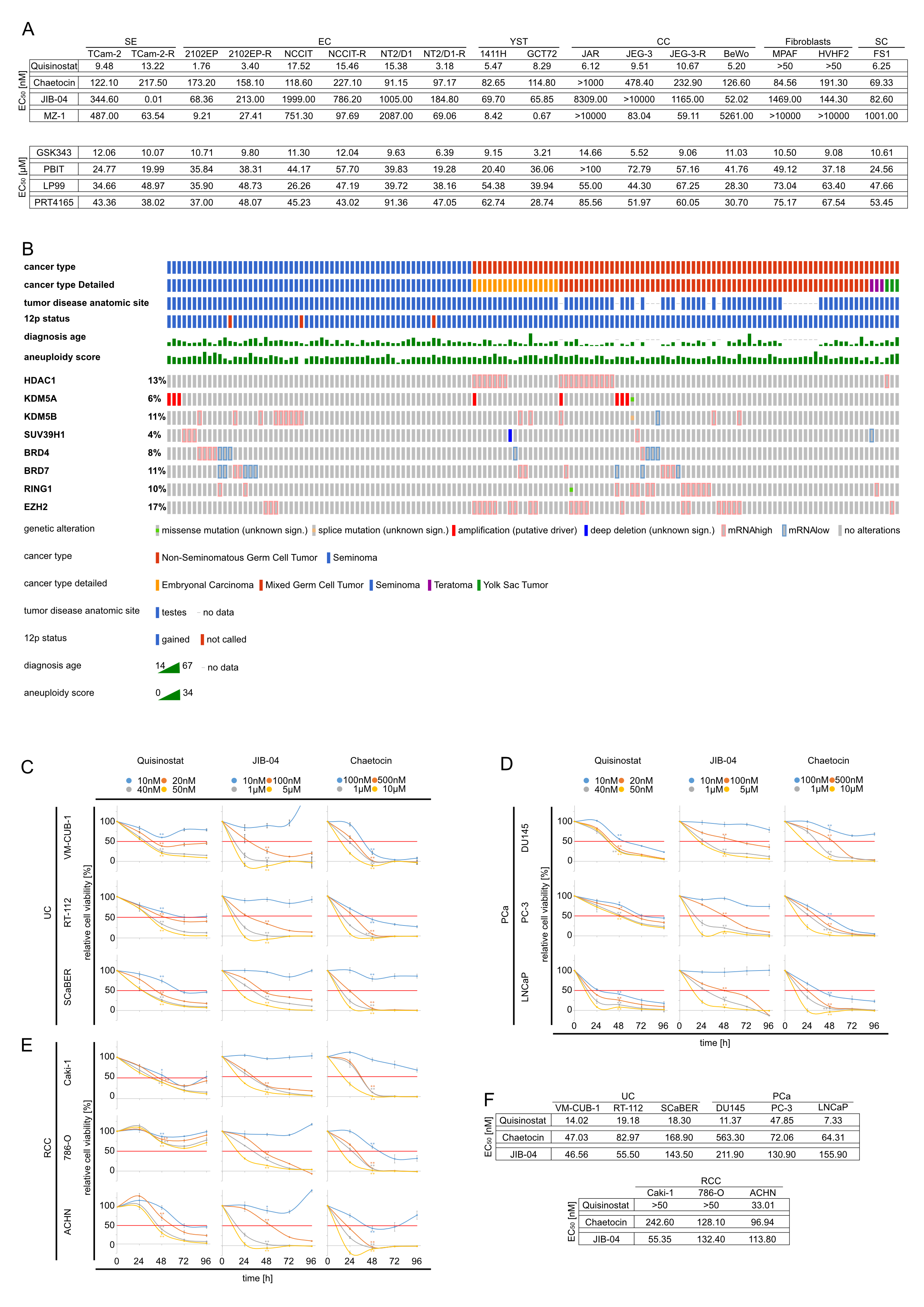

Supplement: Supplementary file 4 — Additional file 4: Figure S4. Epi-drugs decrease cell viability in GCTs and other urological entities. A EC50 values for each GCT cell line, fibroblasts and Sertoli cells and epi-drug treatment were calculated from data shown in Fig. S3. B Analysis of the mutational and gene expression alteration spectrum of the top eight target genes using the cBioPortal tool on the 'Testicular Germ Cell Tumors' cohort of TCGA. C–E XTT data for Quisinostat, JIB-04 and Chaetocin treatment in three cell lines each for UC (C), PCa (D) and RCC (E). The red line indicates 50% cell viability. Asterisks indicate significant changes between treatment and solvent control (after 48 h, time point chosen for EC50 calculation) (*p < 0.05, **p < 0.005). F EC50 values from (C), (D) and (E) calculated using GraphPad Prism v8. EC50: half-maximal effective concentration where the cell viability has 50% compared to the solvent control. SE, seminoma; EC, embryonal carcinoma; YST, yolk-sac tumor; CC, choriocarcinoma; SC, Sertoli cells; UC, urothelial carcinoma; PCa, prostate cancer; RCC, renal cell carcinoma. [file 13148_2021_1223_MOESM4_ESM.tiff]

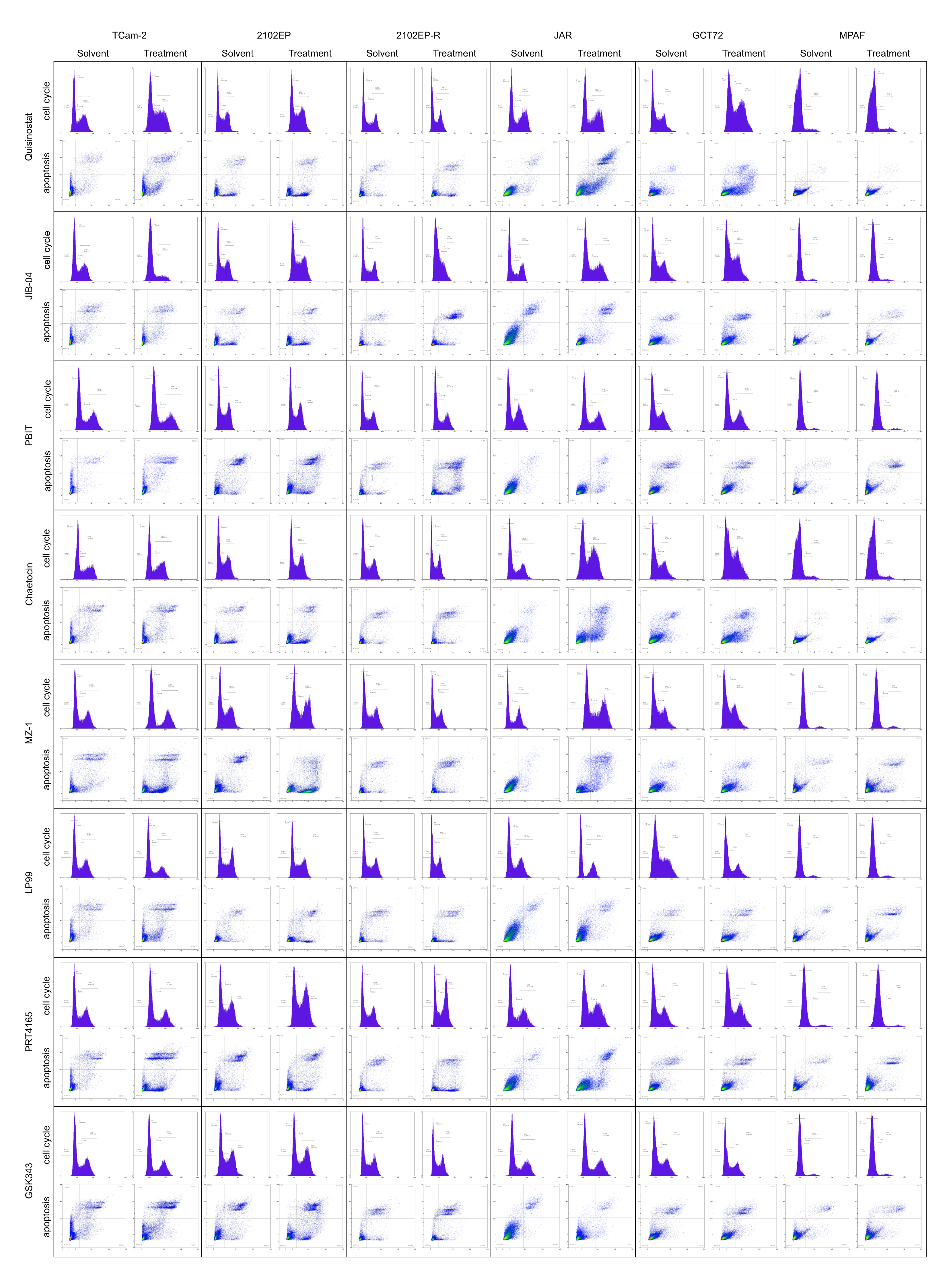

Supplement: Supplementary file 5 — Additional file 5: Figure S5. Raw data of flow cytometry analysis of Annexin V/PI (apoptosis) or PI staining only (cell cycle) of indicated cell lines (columns) after 16 h of treatment with indicated epi-drugs (rows). [file 13148_2021_1223_MOESM5_ESM.tiff]

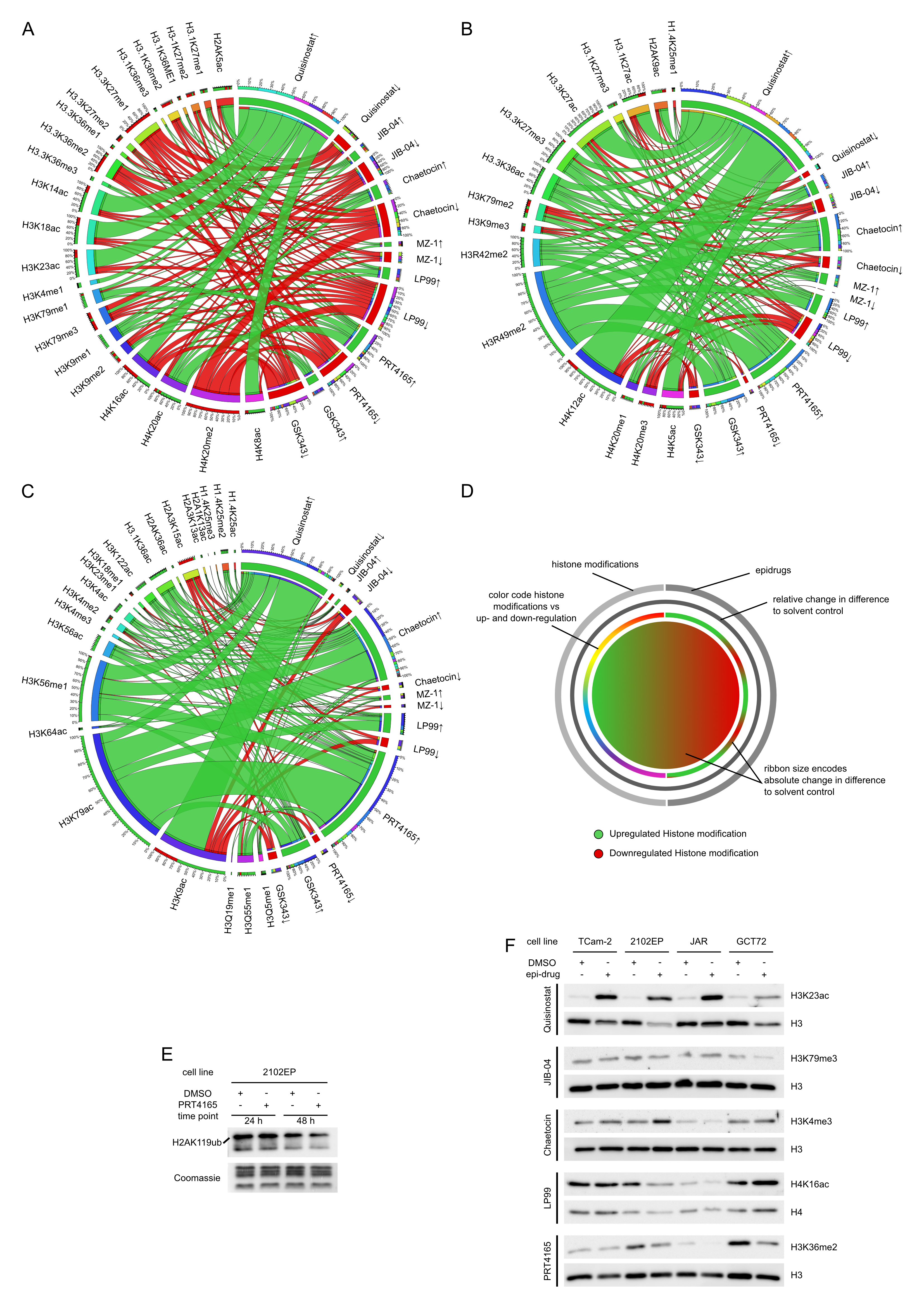

Supplement: Supplementary file 6 — Additional file 6: Figure S6. Mass spectrometry analysis of histone modifications arranged by frequency. A–C Circos diagrams are illustrating the difference in peptide frequency of given histone modifications of treated 2102EP cells compared to the solvent control. Histone modifications with a general peptide frequency of 10–100% (A), 1–10% (B) and 0–1% (C) are depicted separately for better comparison. Only differences in modification abundancy > 1 (10–100%), > 0.1 (1–10%) or > 0.01 (0.1–1%) were considered. D Depiction of the information given by the circos diagram rings. E Western blot analysis of histone extracts from PRT4165 or solvent control DMSO treated 2102EP cells after 24 h and 48 h. Coomassie staining is shown as loading control. F Western blot analysis of selected histone modifications in GCT cell lines. Cells were treated with indicated epi-drugs for 16 h. Total H3/H4 was used as loading control. ↑: upregulation, ↓: downregulation. [file 13148_2021_1223_MOESM6_ESM.tiff]

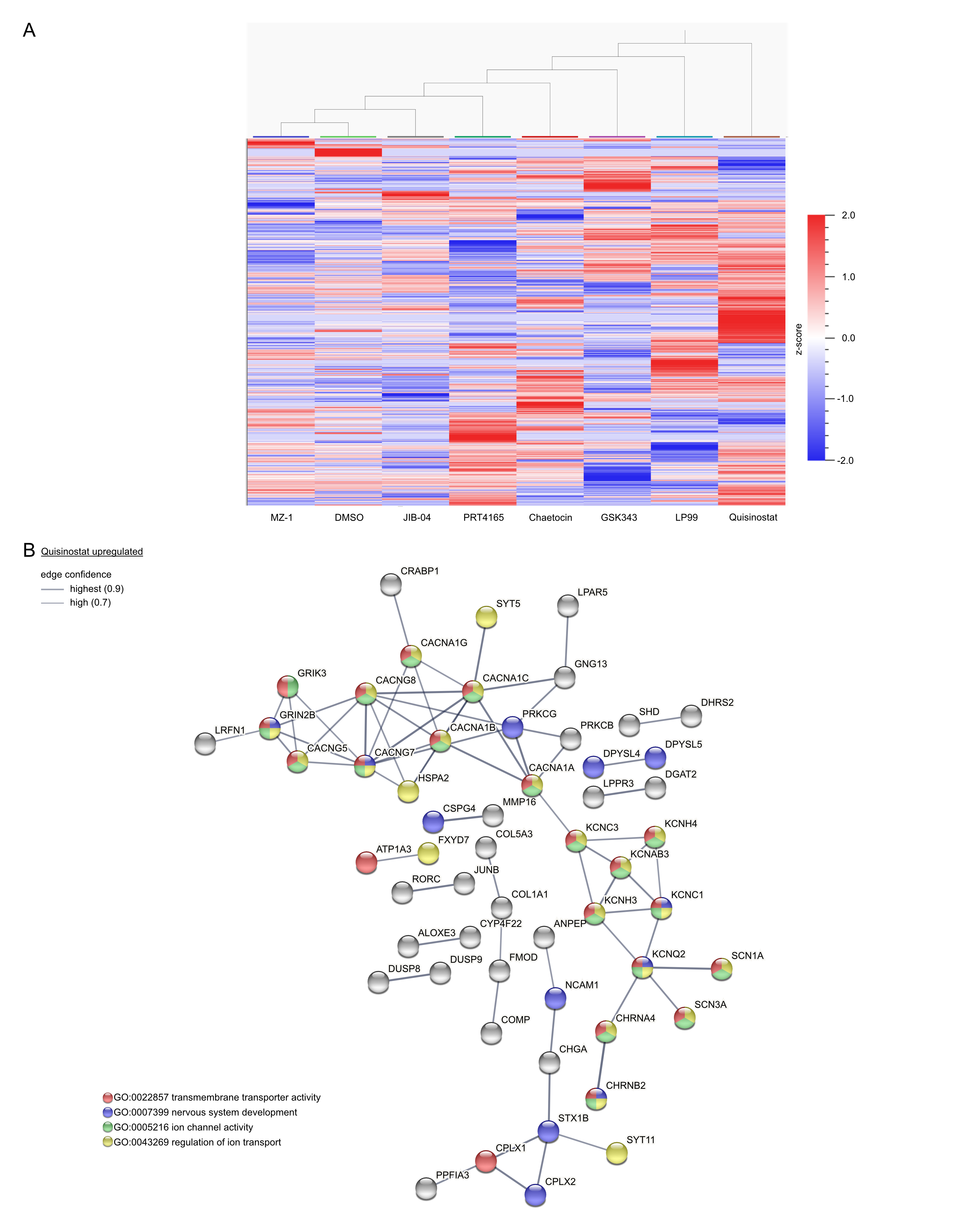

Supplement: Supplementary file 7 — Additional file 7: Figure S7. A Unsupervised clustering heatmap of RNA seq data (DMSO control n = 3, epi-drugs n = 1). 2102EP were treated with indicated epi-drugs for 16 h. B STRING analysis of upregulated genes in Quisinostat treated (16 h) 2102EP cells. [file 13148_2021_1223_MOESM7_ESM.tiff]

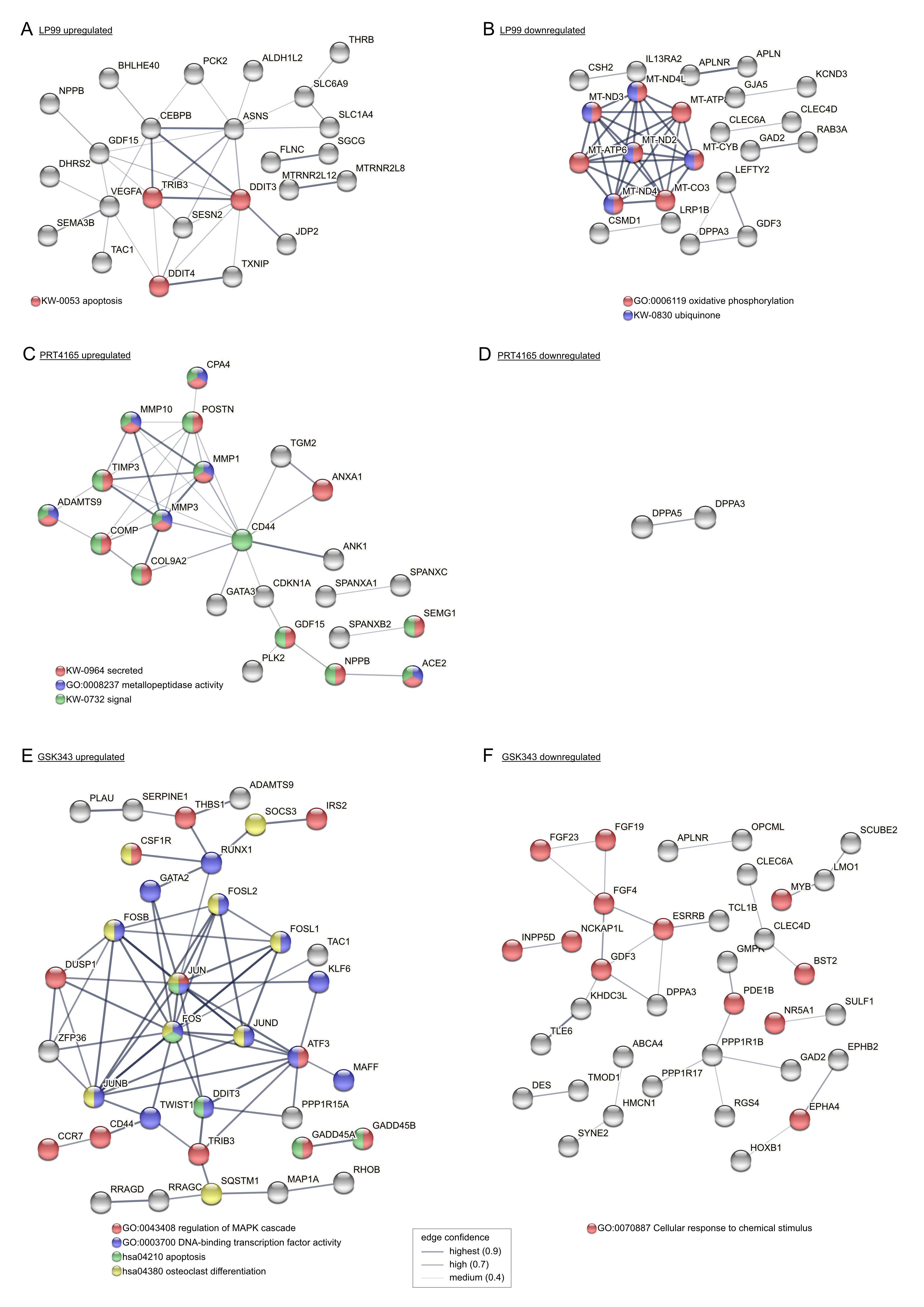

Supplement: Supplementary file 8 — Additional file 8: Figure S8. STRING analysis of deregulated genes after 16 h of indicated epi-drug treatments in 2102EP cells (DMSO control n = 3, epi-drugs n = 1). A LP99 upregulated genes, B LP99 downregulated genes, C PRT4165 upregulated genes, D PRT4165 downregulated genes, E GSK343 upregulated genes, F GSK343 downregulated genes. [file 13148_2021_1223_MOESM8_ESM.tiff]

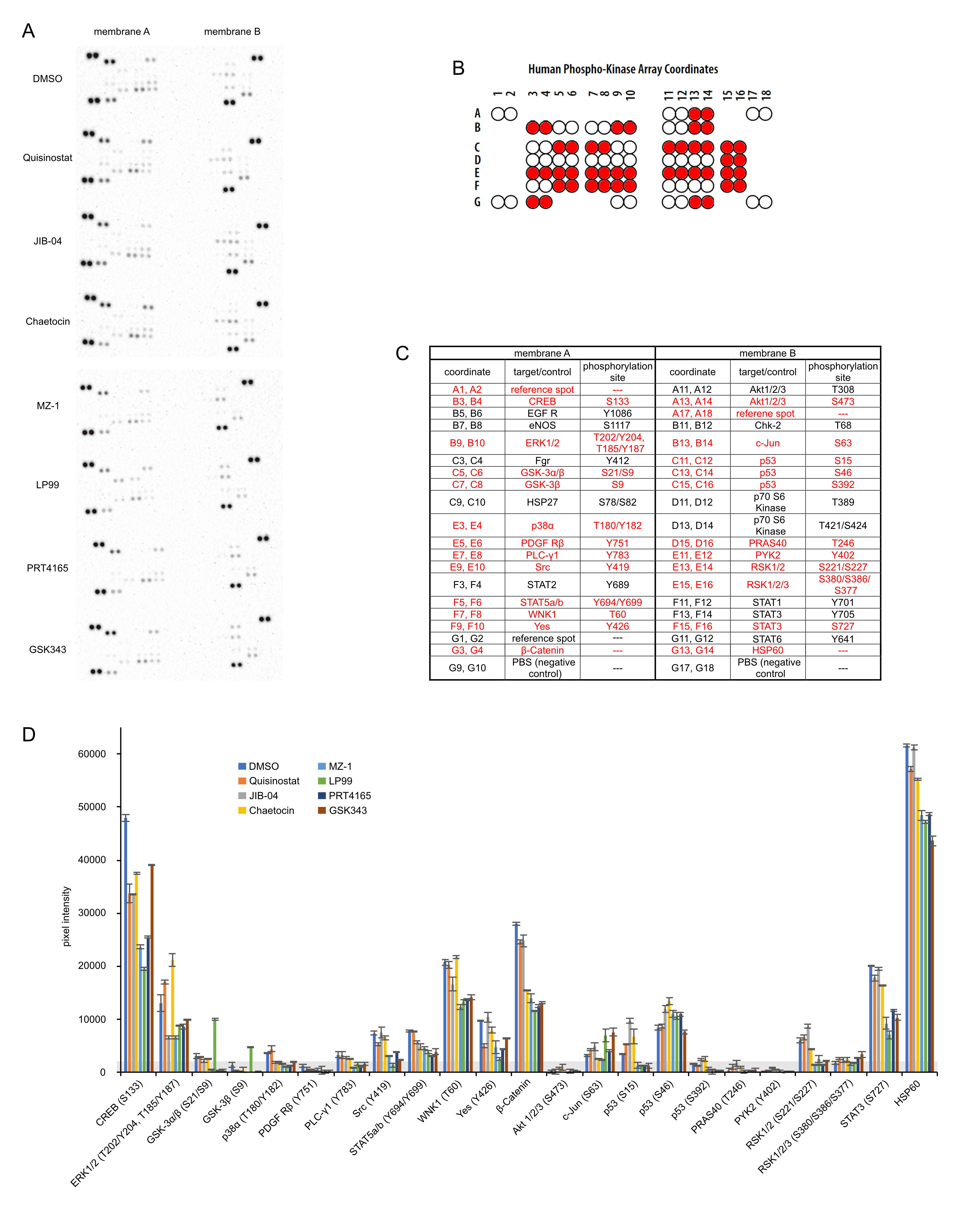

Supplement: Supplementary file 9 — Additional file 9: Figure S9. A Proteome Profiler Human Phospho-Kinase Array raw data. 2102EP were treated with indicated epi-drugs for 16 h (n = 2). B Membrane layout of kinase array with corresponding spotted antibodies given in C. Detectable dots (marked in red) were used for quantification with ImageJ as shown in D. Gray bar indicates cut-off value of 2500. [file 13148_2021_1223_MOESM9_ESM.tiff]

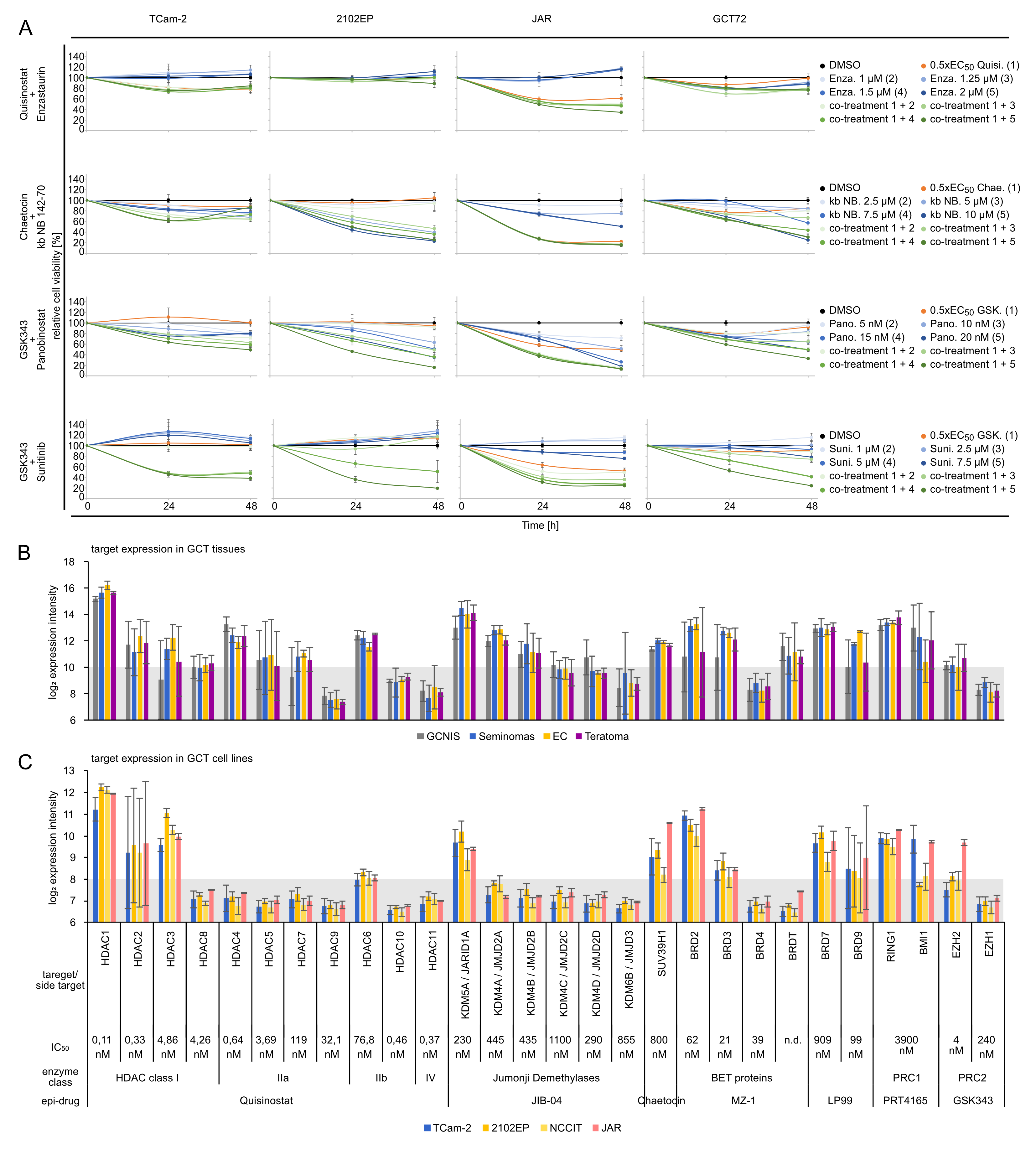

Supplement: Supplementary file 10 — Additional file 10: Figure S10. A XTT cell viability raw data of co-treatments. Cells were pretreated with the 0.5 × EC50 concentration of each epi-drug for 24 h, followed by treatment with different concentrations of the co-drug for 24 h and 48 h. B, C Epi-drug side target gene expression in GCT tissues (B) (normal testis tissue (n = 4), GCNIS (n = 3), SE (n = 4), EC (n = 3), TE (n = 3) and mixed non-seminomas (n = 4) and cell lines (C) (TCam-2 (n = 5), 2102EP (n = 5), NCCIT (n = 4) and JAR (n = 2). Expression microarray data were re-analyzed in context of this study [10, 15, 16, 19–22, 90, 96]. IC50 values from published data for Quisinostat [23], JIB-04 [35], Chaetocin [31], MZ-1 [103], LP99 [36], PRT4165 [27] and GSK343 [34]. GCT, germ cell tumors; GCNIS, germ cell neoplasia in situ; EC, embryonal carcinoma; IC50, half-maximal inhibitory concentration; HDAC, histone deacetylase; BET, bromodomain and extra-terminal motif; PRC, poly repressive complex. [file 13148_2021_1223_MOESM10_ESM.tiff]
